# Supplementary material for: Coinfections by noninteracting pathogens are not independent and require new tests of interaction
Source: PLoS Biol. 2019 Dec 3;17(12):e3000551. doi: 10.1371/journal.pbio.3000551 (PMC6890165; doi:10.1371/journal.pbio.3000551)
Supplement: S1 Text — Further mathematical details on the models considered in the main text, as well as showing how the models can be extended to account for pathogen-specific rates of clearance. (PDF) [file pbio.3000551.s001.pdf]

S1 Text. Mathematical supplements

Organisation of this document

This document consists of six sections.

- 1. "The proportion of co-infected hosts is larger than the product of the prevalences".
  - A demonstration that $J_{1,2}$ from Eq. (3) in the Main Text is larger than $P = I_1 I_2$ as can be derived from Eq. (1) in the Main Text for all parameters (for sufficiently large t), as well as numerical work to investigate how quickly this is likely to occur.
- 2. "Covariance matrix at the endemic equilibrium".
  - A derivation of the covariance matrix for stochastic fluctuations around the endemic equilibrium in the two-pathogen model (leads to Eq. (27) in the main text).
- 3. "Comments on the model of May and Nowak (1995)".
  - Details how the oft-cited co-infection model of May and Nowak (1995) is not correct.
- 4. "Extending the models to accommodate specific clearance".
  - Shows how the methods developed in the main text can be extended to accommodate an additional epidemiological parameter: specific clearance (i.e. each pathogen being cleared independently of any other, possibly at a pathogen-specific rate).
- 5. "The prevalence of co-infections can be equal to the product of the prevalences of interacting pathogens".
  - Shows how, in the model described in S1 Text Section 4, if there is no host natural death but only specific clearance, then the prevalence of co-infections can be equal to the product of the prevalences even when pathogens interact. This complements our main point (non-interaction does not imply independence) by showing that it holds the other way around as well (independence does not imply non-interaction).
- 6. "Impact of environmental noise and transient behaviour".
  - Shows how, in the two-pathogen model considered in the main text, the difference between $J_{1,2}$ and P is robust to the form of the noise that is assumed, as well as that it is likely to become detectable relatively quickly and so that transient behaviour is not likely to critically affect our results.

1 The proportion of co-infected hosts is larger than the product of the prevalences

1.1 Mathematical proof of long-term behaviour

Let $P = I_1 I_2$ and introduce the new variable Z, the extent to which the product of prevalences over-estimates the proportion of co-infected hosts,

$$Z = P - J_{1,2} = I_1 I_2 - J_{1,2}. \tag{S1}$$

Differentiating Z and simplifying using Eqs. (2) and (3) in the main text leads to

$$\begin{aligned} \dot{Z} &= I_1 \dot{I}_2 + I_2 \dot{I}_1 - \dot{J}_{1,2}, \\ \dot{Z} &= -(\beta_1 I_1 + \beta_2 I_2 + \mu) Z - \mu P. \end{aligned} \tag{S2}$$

Of interest is the sign of $Z(t)$ as a function of its initial conditions. We assume $R_{0,i} > 1$ and $0 < I_i(0) \leq \bar{I}_i$ for $i = 1, 2$. The differential equation for I_i is logistic and therefore, $I_i(t)$ converges monotonically to $\bar{I}_i$, $i = 1, 2$. There exists $\epsilon > 0$ such that $P(t) > \tilde{P} = \bar{I}_1 \bar{I}_2 - \epsilon > 0$ for $t \geq 0$. Suppose $Z(0) > 0$. The term $-\mu P(t) < -\mu \tilde{P} < 0$ ensures that $Z(t)$ decreases to zero in finite time. Let t_s be the first time that $Z(t_s) = 0$. Then $\dot{Z}(t_s) < 0$, so $Z(t)$ eventually becomes negative. To show that $Z(t)$ remains negative, let $Z(t) < 0$ for $t_s < t < t_2$ and let t_2 be the first time that $Z(t_2) = 0$, then $\dot{Z}(t_2) \geq 0$, a contradiction to the fact that $\dot{Z}(t_2) \leq -\mu \tilde{P}$. Hence $Z(t)$ remains negative for $t > t_s$. In a similar manner it can be shown that if $Z(0) = 0$ or $Z(0) < 0$, then $Z(t) < 0$ for $t > 0$. In particular, due to the convergence of $I_i(t)$ to $\bar{I}_i$ for $i = 1, 2$, $Z(t)$ converges to the negative limit: $-\mu \bar{I}_1 \bar{I}_2 / (\beta_1 \bar{I}_1 + \beta_2 \bar{I}_2 + \mu)$.

1.2 Numerical investigation of the switching time, t_s

That $Z(t)$ remains negative after any change of sign is proved above. The "switching time" at which this occurs, t_s, then becomes of potential interest. It seems rather difficult to come to any strong conclusions analytically. We therefore performed a numerical investigation of the switching time, summarising the behaviour of the two-pathogen model for different sets of initial conditions and parameters to understand the timing of this event.

In particular, we simulated our deterministic, two-pathogen model a very large number of times (100,000 for each initial condition scenario; see below) with randomly-chosen values of the epidemiological parameters. In each run of the model, the infection rates β_1 and β_2 were sampled uniformly at random between 1 and 5, keeping the removal rate, μ, fixed at 1 (i.e. scaling all simulations relative to the same host lifetime). This allows us to characterise behaviour for $1 \leq R_{0,1}, R_{0,2} \leq 5$.

We identified two sets of initial conditions that might be of interest.

1. **Random initial conditions.** The initial condition for all four host densities in our model were chosen uniformly at random, subject to the constraint that $J_{\varnothing}(0) + J_1(0) + J_2(0) + J_{1,2}(0) = 1$ (this was done by sampling from a suitable Dirichlet distribution). This corresponds to the case in which nothing is known about the initial state of the system.

2. **One pathogen is invading.** The initial condition for the density of hosts singly infected by pathogen one, $J_1(0)$, was fixed at its equilibrium value in the absence of the other pathogen (i.e. $J_1(0) = 1 - 1/R_{0,1}$) and the initial density of co-infected hosts, $J_{1,2}(0)$ was fixed at 0. The density of hosts singly infected

by pathogen two, $J_2(0)$, was then sampled uniformly at random within a range of "small" values (the maximum permissible value of $J_2(0)$ was chosen to be 0.01). The density of susceptible hosts was then set to be $J_\varnothing(0) = 1/R_{0,1} - J_2(0)$, again to ensure the total density of hosts was fixed at 1. This scenario corresponds to pathogen two invading pathogen one when it is initially present at its equilibrium.

Under both initial condition scenarios, the switching time was relatively small. Typical examples showing how the switching time was calculated are shown in S1 Fig (panels A and C). For the random initial condition scenario, in approximately 50% of cases the initial conditions were such that $Z < 0$ already at $t = 0$ (in which case the switch does not occur, with Z remaining negative forever). In the remaining 50% of cases, the switching time almost always satisfied $t_s < 0.5$ (host lifetimes), and was very often far shorter (S1B Fig). In the scenario corresponding to one pathogen invading the other, the initial value of Z was always positive, and very short switching times were not possible. Nevertheless, in all cases we tested, the switch occurred within the average lifetime of a single host.

1.3 Summary

In summary, the fate of Z is to become negative in finite time and to remain negative thereafter. This is due to $\mu > 0$. Otherwise for $\mu = 0$, Z would not change sign and would asymptotically converge to zero. The timing of the switch from positive to negative values of Z – if it is even applicable, which depends on the initial conditions – depends on parameters and initial conditions, but is always relatively fast compared to the average lifetime of an individual host.

2 Covariance matrix at the endemic equilibrium

In the stochastic version of the model, the fluctuations $(\Delta I_1, \Delta I_2) = (I_1 - \bar{I}_1, I_2 - \bar{I}_2)$ about the endemic equilibrium $(\bar{I}_1, \bar{I}_2) = N(1-1/R_{0,1}, 1-1/R_{0,2})$ can be approximated by the solution of the linear multivariate Fokker-Planck equation,

$$\frac{\partial p(x,t)}{\partial t} = -\sum_{i,j=1}^{2} A_{ij} \frac{\partial (x_j p)}{\partial x_i} + \frac{1}{2} \sum_{i,j=1}^{2} B_{ij} \frac{\partial^2 p}{\partial x_i \partial x_j}, \tag{S3}$$

where the vector $x = (x_1, x_2)$ corresponds to $(\Delta I_1, \Delta I_2)$. The steady-state solution of this equation is a Gaussian distribution with mean zero and covariance matrix $\bar{C}$. We will use this multivariate normal distribution to approximate the joint probability density function of the random variables (I_1, I_2) near the endemic equilibrium. Matrix $A = [A_{ij}]$ is the rate of change toward zero and matrix $B = [B_{ij}]$ is the covariance of this process (O'Dea et al., 2018; Van Kampen, 1992). In particular, matrix A is the linearization of the differential equations for (I_1, I_2) (total population size, not proportions) about the endemic equilibrium,

$$A = \begin{bmatrix} \beta_1 - 2\frac{\beta_1}{N}\bar{I}_1 - \mu & 0 \\ 0 & \beta_2 - 2\frac{\beta_2}{N}\bar{I}_1 - \mu \end{bmatrix} = \begin{bmatrix} -\beta_1 + \mu & 0 \\ 0 & -\beta_2 + \mu \end{bmatrix}. \tag{S4}$$

We use the fact that $I_1 = J_1 + J_{1,2}$ and $I_2 = J_2 + J_{1,2}$ and sum the appropriate elements in the covariance matrix Σ (Eq. (24) in the main text) to compute the covariance

matrix B,

$$B=\begin{bmatrix} \bar{F}_1(\bar{J}_\varnothing+\bar{J}_2)+\mu\bar{I}_1 & \mu\bar{J}_{12} \\ \mu\bar{J}_{12} & \bar{F}_2(\bar{J}_\varnothing+\bar{J}_1)+\mu\bar{I}_2 \end{bmatrix}=\begin{bmatrix} 2N\mu\left(1-\frac{1}{R_{0,1}}\right) & \mu\bar{J}_{12} \\ \mu\bar{J}_{12} & 2N\mu\left(1-\frac{1}{R_{0,2}}\right) \end{bmatrix}. \quad (S5)$$

The expressions in matrices A and B are evaluated at the endemic equilibrium. In particular, $\bar{F}_i = \beta_i\bar{I}_i/N = \beta_i(1-1/R_{0,i})$, $i = 1,2$, and the equilibrium $\bar{J}_{12}$ is found by multiplying Eq. (4) in the main text by N and substituting for the equilibria of the single pathogen models $\bar{I}_1$ and $\bar{I}_2$:

$$\bar{J}_{12}=\left(\frac{N(\beta_1+\beta_2)}{\beta_1+\beta_2-\mu}\right)\left(1-\frac{1}{R_{0,1}}\right)\left(1-\frac{1}{R_{0,2}}\right). \quad (S6)$$

Van Kampen (1992) showed that the covariance matrix C of the Fokker-Planck equation is the solution of the differential equation: $\dot{C} = AC + CA^T + B$. The steady-state covariance matrix is the solution of

$$AC+CA^T=-B\,. \quad (S7)$$

To compute the steady-state covariance matrix for the proportion of the population that is infected, divide the solution of Eq. (S7) by N^2. That is, $\bar{C}$ equals

$$\bar{C}=\frac{1}{N^2}\begin{bmatrix} \frac{N\mu\left(1-\frac{1}{R_{0,1}}\right)}{\beta_1-\mu} & \frac{\mu\bar{J}_{1,2}}{(\beta_1-\mu)+(\beta_2-\mu)} \\ \frac{\mu\bar{J}_{1,2}}{(\beta_1-\mu)+(\beta_2-\mu)} & \frac{N\mu\left(1-\frac{1}{R_{0,2}}\right)}{\beta_2-\mu} \end{bmatrix}=\begin{bmatrix} \frac{1}{NR_{0,1}} & \frac{\mu\bar{J}_{1,2}}{N^2[(\beta_1-\mu)+(\beta_2-\mu)]} \\ \frac{\mu\bar{J}_{1,2}}{N^2[(\beta_1-\mu)+(\beta_2-\mu)]} & \frac{1}{NR_{0,2}} \end{bmatrix}, \quad (S8)$$

where $\bar{J}_{12}$ is defined in Eq. (S6). The steady-state covariance matrix in Eq. (S8) is used to construct confidence ellipses about the endemic equilibrium $(\bar{I}_1/N, \bar{I}_2/N) = (1-1/R_{0,1}, 1-1/R_{0,2})$ (as shown in Fig. 2C in the main text).

Note that the covariance between the prevalences of pathogen 1 and pathogen 2 (the off-diagonal elements in Eq. (S8)) is

$$\bar{C}_{ij}=\mathrm{cov}\left(\frac{I_1}{N},\frac{I_2}{N}\right)=\frac{\mu\bar{J}_{1,2}}{N^2[(\beta_1-\mu)+(\beta_2-\mu)]}=\frac{(\beta_1+\beta_2)(\beta_1-\mu)(\beta_2-\mu)\mu}{N\beta_1\beta_2(\beta_1+\beta_2-\mu)(\beta_1-\mu+\beta_2-\mu)}\geq 0\,, \quad (S9)$$

(for $i \neq j$) with equality if and only if $\mu = 0$ (assuming $\beta_i > \mu$, $i = 1,2$).

3 Comments on the model of May and Nowak (1995)

May and Nowak (1995) introduced a co-infection model very similar to that presented in the main text, taking

$$\dot{I}_i=I_i[\beta_i(1-I_i)-\mu-\bar{\alpha}_i]\,,\quad \text{with} \quad i=1,\ldots,n\,, \quad (S10)$$

for n pathogens. The natural mortality rate of the host is μ. The only difference from our model is pathogen-specific mortality. In a single infection, pathogen i induces an additional death rate to the host α_i: this is the virulence of pathogen i. The induced death rate of co-infected hosts is assumed to be equal to the maximum virulence of the co-infecting pathogens. The pathogens are ranked such that for all i, $\alpha_i < \alpha_{i+1}$. Pathogen 1 is the least virulent pathogen and n is the most virulent pathogen. The term $\bar{\alpha}_i$ denotes the average induced death rate of hosts infected by pathogen i.

The authors state that the probability that a host is not infected with a pathogen more virulent than i is defined as:

$$p_i = \prod_{j=i+1}^{n} (1 - I_j)\,. \tag{S11}$$

It is important to notice that an underlying assumption of this definition is that the dynamics of the pathogens are independent. But, as we show below, they are not, since the most virulent pathogens influence the dynamics of least virulent pathogens. The coupling term $\bar{\alpha}_i$ is defined as:

$$\bar{\alpha}_i = \alpha_i p_i + \sum_{j=i+1}^{n} \alpha_j I_j p_j\,. \tag{S12}$$

The term $I_j p_j$ represents the probability to be infected by j and uninfected by more virulent pathogens than j. Again, this definition implicitly assumes that the dynamics of the pathogens are independent. This seems to contradict the fact that the dynamics are coupled through virulence.

In this section, we check the model given in Eq. (S10) for $n = 2$ pathogens and show that the above definitions do not hold up to mathematical analysis. We consider the same 2-pathogen model as Eq. (3) of the main text, except that we include additional virulence parameters $\alpha_2 > \alpha_1$. Model (S10) is to be compared with:

$$\begin{aligned}
\dot{J}_1 &= F_1 J_\varnothing - (F_2 + \mu + \alpha_1) J_1\,,\\
\dot{J}_2 &= F_2 J_\varnothing - (F_1 + \mu + \alpha_2) J_2\,,\\
\dot{J}_{1,2} &= F_2 J_1 + F_1 J_2 - (\mu + \max(\alpha_1, \alpha_2)) J_{1,2}\,,\\
&= F_2 J_1 + F_1 J_2 - (\mu + \alpha_2) J_{1,2}\,,
\end{aligned} \tag{S13}$$

where $J_\varnothing = 1 - J_1 - J_2 - J_{1,2}$.

Since model (S10) and model (S13) share the same biological assumptions and the same mathematical formalism, they should be equivalent (for $n = 2$ pathogens). Let $I_1 = J_1 + J_{1,2}$ and $I_2 = J_2 + J_{1,2}$. Model (S13) is equivalent to

$$\begin{aligned}
\dot{I}_1 &= \beta_1 I_1 (1 - I_1) - (\mu + \alpha_1^\star) I_1\,,\\
\dot{I}_2 &= \beta_2 I_2 (1 - I_2) - (\mu + \alpha_2) I_2\,,\\
\dot{J}_{1,2} &= \beta_1 I_1 (I_2 - J_{1,2}) + \beta_2 I_2 (I_1 - J_{1,2}) - (\mu + \alpha_2) J_{1,2}\,,
\end{aligned} \tag{S14}$$

where

$$\alpha_1^\star = \alpha_1 \left(1 - \frac{J_{1,2}}{I_1}\right) + \alpha_2 \frac{J_{1,2}}{I_1}\,. \tag{S15}$$

Eq. (S11) yields $p_1 = 1 - I_2$ and $p_2 = 1$. Eq. (S12) yields

$$\bar{\alpha}_1 = \alpha_1 (1 - I_2) + \alpha_2 I_2\,. \tag{S16}$$

For model (S10) and model (S13) to coincide, one must have $\alpha_1^\star = \bar{\alpha}_1$, i.e. $J_{1,2} = I_1 I_2$. Proceeding as in S1 Text Section 1.1, let $P = I_1 I_2$ and $Z = P - J_{1,2}$. We have

$$\dot{Z} = -(\beta_1 I_1 + \beta_2 I_2 + \mu + \alpha_2) Z - (\mu + \alpha_1^\star) P\,. \tag{S17}$$

Assuming $P(t) > \tilde{P} > 0$ for $t > 0$, it can be shown that $Z(t)$ becomes negative and stays negative, implying for some time t_0 and $t > t_0$, $J_{1,2}(t) > P(t) = I_1(t) I_2(t)$. Therefore, $\alpha_1^\star \neq \bar{\alpha}_1$. Hence, model (S10) and model (S13) are not equivalent, as they should be, if model (S10) is correct.

4 Extending the models to accommodate specific clearance

4.1 Two-pathogen model

Introducing a pathogen-specific clearance rate γ_i, Eq. (1) of the main text is replaced by

$$\dot{I}_i = \beta_i I_i (1 - I_i) - (\gamma_i + \mu) I_i \tag{S18}$$

and Eq. (3) of the main text by

$$\begin{aligned} \dot{J}_1 &= F_1 J_\varnothing - (F_2 + \gamma_1 + \mu) J_1 + \gamma_2 J_{1,2}\,, \\ \dot{J}_2 &= F_2 J_\varnothing - (F_1 + \gamma_2 + \mu) J_2 + \gamma_1 J_{1,2}\,, \\ \dot{J}_{1,2} &= F_2 J_1 + F_1 J_2 - (\gamma_1 + \gamma_2 + \mu) J_{1,2}\,, \end{aligned} \tag{S19}$$

where the definition of F_i is the same as in Eq. (2). The parameter μ is unchanged: this is the natural death rate of the host.

After inclusion of pathogen-specific clearance rates, Eq. (8) of the main text is replaced by

$$\dot{J}_\varnothing = \mu (J_1 + J_2 + J_{1,2}) - (F_1 + F_2) J_\varnothing + \gamma_1 J_1 + \gamma_2 J_2 = \mu (1 - J_\varnothing) - (F_1 + F_2) J_\varnothing + \gamma_1 J_1 + \gamma_2 J_2\,. \tag{S20}$$

and the basic reproduction number is

$$R_{0,i} = \frac{\beta_i}{\gamma_i + \mu}\,. \tag{S21}$$

Also, Eq. (9) of the main text is replaced by

$$\dot{J}_{1,2} = \beta_2 I_2 (I_1 - J_{1,2}) + \beta_1 I_1 (I_2 - J_{1,2}) - (\gamma_1 + \gamma_2 + \mu) J_{1,2}\,. \tag{S22}$$

Eqs. (4) and (5) are unchanged as the relative deviation from statistical independence is unaffected by the specific clearance rates γ_i.

Finally, Eq. (S2) is replaced by

$$\dot{Z} = -(\beta_1 I_1 + \beta_2 I_2 + \gamma_1 + \gamma_2 + \mu) Z - \mu P. \tag{S23}$$

where $Z(t)$ converges to the negative limit: $-\mu \bar{I}_1 \bar{I}_2 / (\beta_1 \bar{I}_1 + \beta_2 \bar{I}_2 + \gamma_1 + \gamma_2 + \mu)$. Again, the fate of Z is to become negative and to remain negative provided $\mu > 0$.

4.2 Analysis of the n-pathogen model

Introducing the notation for the set of hosts infected by one additional pathogen $\Lambda_i = \Gamma \cup \{i\}$ (for $i \notin \Gamma$), Eq. (6) in the main text becomes

$$\dot{J}_\Gamma = \sum_{i \in \Gamma} F_i J_{\Omega_i} - \left(\sum_{i \notin \Gamma} F_i + \sum_{i \in \Gamma} \gamma_i + \mu \right) J_\Gamma + \sum_{i \notin \Gamma} \gamma_i J_{\Lambda_i}\,. \tag{S24}$$

with F_i the same as in Eq. (7). The final term in Eq. (S24) tracks the inflow due to hosts with one additional infection that clear a single infection. This final term is omitted in the single case in which Γ corresponds to infection by all pathogens. Also, the updated version of Eq. (10) for $\dot{J}_\varnothing$ with pathogen-specific clearance rates is

$$\dot{J}_\varnothing = \mu (1 - J_\varnothing) - \left(\sum_{i=1}^{n} F_i \right) J_\varnothing + \sum_{i=1}^{n} \gamma_i J_i\,. \tag{S25}$$

Equilibrium analysis. The equilibrium equations with pathogen-specific clearance rates are

$$0 = \sum_{i\in\Gamma} \bar{F}_i \bar{J}_{\Omega_i} - \left(\sum_{i\notin\Gamma} \bar{F}_i + \sum_{i\in\Gamma} \gamma_i + \mu \right) \bar{J}_\Gamma + \sum_{i\notin\Gamma} \gamma_i \bar{J}_{\Lambda_i}, \tag{S26}$$

and

$$0 = \sum_{i\in\Gamma} (\beta_i - (\gamma_i + \mu)) \bar{J}_{\Omega_i} - \left(\sum_{i\notin\Gamma} (\beta_i - (\gamma_i + \mu)) + \sum_{i\in\Gamma} \gamma_i + \mu \right) \bar{J}_\Gamma + \sum_{i\notin\Gamma} \gamma_i \bar{J}_{\Lambda_i}. \tag{S27}$$

with

$$\bar{F}_i = \beta_i \bar{I}_i = \beta_i \left(1 - \frac{\gamma_i + \mu}{\beta_i} \right) = \beta_i - (\gamma_i + \mu). \tag{S28}$$

(replacing Eqs. (11-12-13) of the main text).

To fit the models to data, it would be necessary to scale by the rate of host natural death μ in Eq. (S27), leading to

$$0 = \sum_{i\in\Gamma} \left(\hat{\beta}_i - (\hat{\gamma}_i + 1)\right) \bar{J}_{\Omega_i} - \left(\sum_{i\notin\Gamma} \left(\hat{\beta}_i - (\hat{\gamma}_i + 1)\right) + \sum_{i\in\Gamma} \hat{\gamma}_i + 1 \right) \bar{J}_\Gamma + \sum_{i\notin\Gamma} \hat{\gamma}_i \bar{J}_{\Lambda_i}, \tag{S29}$$

and so consider infection ($\hat{\beta}_i = \beta_i/\mu$) and specific clearance ($\hat{\gamma}_i = \gamma_i/\mu$) rates measured relative to the rate of host natural death. With the scaled force of infection at equilibrium

$$\hat{F}_i = \hat{\beta}_i - (\hat{\gamma}_i + 1), \tag{S30}$$

then Eq. (S29) can be written as

$$0 = \sum_{i\in\Gamma} \hat{F}_i \bar{J}_{\Omega_i} - \left(\sum_{i\notin\Gamma} \hat{F}_i + \sum_{i\in\Gamma} \hat{\gamma}_i + 1 \right) \bar{J}_\Gamma + \sum_{i\notin\Gamma} \hat{\gamma}_i \bar{J}_{\Lambda_i}. \tag{S31}$$

Given the values of $\hat{\beta}_i$ and $\hat{\gamma}_i$, the 2^n-1 linear equations corresponding to Eq. (S31) can be solved simultaneously with the corresponding equation for the equilibrium density of uninfected hosts (i.e. the scaled version of Eq. (S25)):

$$-1 = -\left(\sum_{i=1}^{n} \hat{F}_i + 1 \right) \bar{J}_\varnothing + \sum_{i=1}^{n} \hat{\gamma}_i \bar{J}_i. \tag{S32}$$

to find all 2^n equilibrium prevalences predicted by the n-pathogen model. However, since the recursive solution presented in the main text (Eq. (16)) is no longer available, the system must be solved using (standard) numerical methods for linear systems of equations.

Worked example. When $n = 3$ there is a total of $2^3 = 8$ classes of hosts, uninfected ($J_\varnothing$), singly-infected (J_1, J_2 and J_3), doubly-infected ($J_{1,2}$, $J_{1,3}$ and $J_{2,3}$) and triply-infected ($J_{1,2,3}$). The equilibrium prevalences can be concatenated into a single vector, given here in lexicographical order

$$\mathbf{v} = \left[\bar{J}_\varnothing, \bar{J}_1, \bar{J}_2, \bar{J}_3, \bar{J}_{1,2}, \bar{J}_{1,3}, \bar{J}_{2,3}, \bar{J}_{1,2,3}\right]^T. \tag{S33}$$

If we define $\mathbf{b}$ as

$$\mathbf{b} = [-1, 0, 0, 0, 0, 0, 0, 0]^T, \tag{S34}$$

then Eq. (S31) and (S32) are equivalent to the system of 8 linear equations

$$H\mathbf{v} = \mathbf{b}, \tag{S35}$$

in which matrix H equals

$$\begin{bmatrix} -(\hat{F}_1+\hat{F}_2+\hat{F}_3+1) & \hat{\gamma}_1 & \hat{\gamma}_2 & \hat{\gamma}_3 & 0 & 0 & 0 & 0 \\ \hat{F}_1 & -(\hat{F}_2+\hat{F}_3+\hat{\gamma}_1+1) & 0 & 0 & \hat{\gamma}_2 & \hat{\gamma}_3 & 0 & 0 \\ \hat{F}_2 & 0 & -(\hat{F}_1+\hat{F}_3+\hat{\gamma}_2+1) & 0 & \hat{\gamma}_1 & 0 & \hat{\gamma}_3 & 0 \\ \hat{F}_3 & 0 & 0 & -(\hat{F}_1+\hat{F}_2+\hat{\gamma}_3+1) & 0 & \hat{\gamma}_1 & \hat{\gamma}_2 & 0 \\ 0 & \hat{F}_2 & \hat{F}_1 & 0 & -(\hat{F}_3+\hat{\gamma}_1+\hat{\gamma}_2+1) & 0 & 0 & \hat{\gamma}_3 \\ 0 & \hat{F}_3 & 0 & \hat{F}_1 & 0 & -(\hat{F}_2+\hat{\gamma}_1+\hat{\gamma}_3+1) & 0 & \hat{\gamma}_2 \\ 0 & 0 & \hat{F}_3 & \hat{F}_2 & 0 & 0 & -(\hat{F}_1+\hat{\gamma}_2+\hat{\gamma}_3+1) & \hat{\gamma}_1 \\ 0 & 0 & 0 & 0 & \hat{F}_3 & \hat{F}_2 & \hat{F}_1 & -(\hat{\gamma}_1+\hat{\gamma}_2+\hat{\gamma}_3+1) \end{bmatrix}.$$

The equilibrium prevalence of hosts infected by any combination of pathogens can then be obtained by solving Eq. (S35) for $\mathbf{v}$.

Proof that there is always a unique equilibrium. For the case $n = 3$ pathogens, the matrix $-H$ has off-diagonal entries that are non-positive and diagonal entries that are strictly positive. In addition, the absolute value of each diagonal entry is strictly greater than the absolute value of the sum of all of the other entries in that column. These properties of $-H$ make it a non-singular M-matrix. (Properties of an M-matrix are given in (Plemmons, 1977)). As a consequence of these properties, $-H^{-1}$ exists and is a non-negative matrix from which it follows that the solution $\mathbf{v}$ in Eq. (S35) is non-negative and can be expressed as

$$\mathbf{v} = H^{-1}\mathbf{b}. \tag{S36}$$

Generalizing to the case of n pathogens, it can be verified that matrix $-H$ in Eq. (S35) will still have the same properties, making it a non-singular M-matrix and therefore, the equilibrium $\mathbf{v}$ is the unique non-negative solution given by Eq. (S36).

4.3 Relationship between the NiDP and multinomial models

In this subsection, we show that the equilibrium prevalences in the NiDP model with $\mu = 0$ are equal to the expectations under statistical independence, i.e.,

$$\bar{J}_\Gamma = \prod_{i\in\Gamma} \bar{I}_i \prod_{j\notin\Gamma} (1-\bar{I}_j)\,, \tag{S37}$$

where $\bar{I}_i = 1-\gamma_i/\beta_i$ for all $i \in \{1,2,\ldots,n\}$. In other words, when there is no host natural death (at the timescale of an infection), the probability to be infected by a set of pathogens Γ follows a multinomial distribution with parameters n (the number of distinct pathogens) and $p_i = \bar{I}_i$ for all $i \in \{1,2,\ldots,n\}$.

In the specific case $\mu = 0$, Eq. (S26) becomes

$$0 = \sum_{i\in\Gamma} \bar{F}_i \bar{J}_{\Omega_i} - \left(\sum_{i\notin\Gamma} \bar{F}_i + \sum_{i\in\Gamma} \gamma_i \right) \bar{J}_\Gamma + \sum_{i\notin\Gamma} \gamma_i \bar{J}_{\Lambda_i}\,, \tag{S38}$$

with $\bar{F}_i = \beta_i - \gamma_i$. Eq. (S37) implies

$$\bar{J}_{\Omega_i} = \bar{J}_\Gamma \frac{1-\bar{I}_i}{\bar{I}_i}\,, \quad \text{and} \quad \bar{J}_{\Lambda_i} = \bar{J}_\Gamma \frac{\bar{I}_i}{1-\bar{I}_i}\,. \tag{S39}$$

Substituting the values in Eq. (S39) into the right side of Eq. (S38),

$$\sum_{i\in\Gamma} \left(\bar{F}_i \frac{1-\bar{I}_i}{\bar{I}_i} \right) - \left(\sum_{i\notin\Gamma} \bar{F}_i + \sum_{i\in\Gamma} \gamma_i \right) + \sum_{i\notin\Gamma} \gamma_i \frac{\bar{I}_i}{1-\bar{I}_i} \tag{S40}$$

and simplifying leads to

$$\sum_{i\in\Gamma}\left((\beta_i-\gamma_i)\frac{\gamma_i}{\beta_i-\gamma_i}\right)-\sum_{i\notin\Gamma}(\beta_i-\gamma_i)+\sum_{i\in\Gamma}\gamma_i+\sum_{i\notin\Gamma}\gamma_i\frac{\beta_i-\gamma_i}{\gamma_i}=0.$$

Therefore, the values in Eq. (S37) are equilibrium values.

Similarly, in the specific case $\mu=0$, the equilibrium value for $J_\varnothing>0$ in Eq. (S25) satisfies

$$0=-\left(\sum_{i=1}^{n}\bar{F}_i\right)\bar{J}_\varnothing+\sum_{i=1}^{n}\gamma_i\bar{J}_i\,. \tag{S41}$$

Applying Eq. (S39) and dividing by $\bar{J}_\varnothing$ in the right side of the preceding equation yields

$$\begin{aligned}-\left(\sum_{i=1}^{n}\bar{F}_i\right)+\sum_{i=1}^{n}\gamma_i\frac{\bar{I}_i}{1-\bar{I}_i} &= -\left(\sum_{i=1}^{n}(\beta_i-\gamma_i)\right)+\sum_{i=1}^{n}\gamma_i\frac{\beta_i-\gamma_i}{\gamma_i}\}, &\text{(S42)}\\ &= 0\,. &\text{(S43)}\end{aligned}$$

Hence, Eq. (S37) is the equilibrium solution of the NiDP model in the specific case $\mu=0$.

4.4 Relationship between the NiDP and NiSP models

Returning to the case in which mortality is non-negligible and assuming all pathogens are interchangeable, Eq. (17) of the main text can be replaced by

$$0=|\Gamma|\hat{F}\bar{J}_{\Omega_i}-\big((n-|\Gamma|)\hat{F}+|\Gamma|\hat{\gamma}+1\big)\bar{J}_\Gamma+(n-|\Gamma|)\hat{\gamma}\bar{J}_{\Lambda_i}, \tag{S44}$$

in which

$$\hat{F}=\hat{\beta}-(\hat{\gamma}+1)\,, \tag{S45}$$

(recall the definition of the scaled variables with $\hat{\beta}=\beta/\mu$ and $\hat{\gamma}=\gamma/\mu$).

For $1\le k<n$, substituting Eq. (19) into Eq. (S44) leads to

$$0=k\hat{F}\frac{\bar{M}_{k-1}}{C^n_{k-1}}-\big((n-k)\hat{F}+k\hat{\gamma}+1\big)\frac{\bar{M}_k}{C^n_k}+(n-k)\hat{\gamma}\frac{\bar{M}_{k+1}}{C^n_{k+1}}. \tag{S46}$$

Noting that

$$\frac{C^n_{k+1}}{C^n_{k-1}}=\frac{(n-k+1)(n-k)}{(k+1)k}\quad\text{and}\quad\frac{C^n_{k+1}}{C^n_k}=\frac{n-k}{k+1},$$

it follows that

$$0=(n-k+1)\hat{F}\bar{M}_{k-1}-\big((n-k)\hat{F}+k\hat{\gamma}+1\big)\bar{M}_k+(k+1)\hat{\gamma}\bar{M}_{k+1}, \tag{S47}$$

which holds for $1\le k<n$ (i.e. there is a total of $n-1$ such equations).

When $k=n$ the analogue of Eq. (S46) is

$$0=n\hat{F}\frac{\bar{M}_{n-1}}{C^n_{n-1}}-(n\hat{\gamma}+1)\frac{\bar{M}_n}{C^n_n},$$

and so, since $C^n_{n-1}=n$ and $C^n_n=1$, it follows that

$$0=\hat{F}\bar{M}_{n-1}-(n\hat{\gamma}+1)\bar{M}_n. \tag{S48}$$

When $k = 0$ the analogue of Eqn. (S46) obtained by substituting Eq. (20) into Eq. (S32), is

$$-\left(n\hat{F}+1\right)\frac{\bar{M}_0}{C_0^n}+n\hat{\gamma}\frac{\bar{M}_1}{C_1^n}=-1, \tag{S49}$$

and so, since $C_1^n = n$ and $C_0^n = 1$, it follows that

$$-\left(n\hat{F}+1\right)\bar{M}_0+\hat{\gamma}\bar{M}_1=-1\,. \tag{S50}$$

Taken together, Eqs. (S47-S48-S50) constitute a system of $n+1$ linear equations that fix the equilibrium prevalences of hosts infected by any number of distinct pathogens in the NiSP model.

Worked example. When $n = 3$ there is a total of $n+1 = 4$ classes of host: uninfected (M_0), singly-infected (M_1), doubly-infected (M_2) and triply-infected (M_3). The equilibrium prevalences can be concatenated into a single vector

$$\mathbf{v}=\left[\bar{M}_0,\bar{M}_1,\bar{M}_2,\bar{M}_3\right]^T. \tag{S51}$$

If we define $\mathbf{b}$ as

$$\mathbf{b}=[-1,0,0,0]^T, \tag{S52}$$

then Eq. (S47-S48-S50) are equivalent to the system of 4 linear equations

$$H\mathbf{v}=\mathbf{b}, \tag{S53}$$

in which matrix H equals

$$\begin{bmatrix} -(3F+1) & \hat{\gamma} & 0 & 0 \\ 3\hat{F} & -(2\hat{F}+\hat{\gamma}+1) & 2\hat{\gamma} & 0 \\ 0 & 2\hat{F} & -(\hat{F}+2\hat{\gamma}+1) & 3\hat{\gamma} \\ 0 & 0 & \hat{F} & -(3\hat{\gamma}+1) \end{bmatrix}. \tag{S54}$$

The equilibrium prevalences of hosts infected by any number of distinct pathogens can then be obtained by solving Eq. (S53).

4.5 Relationship between the NiSP and binomial models

In this subsection, we show that the equilibrium prevalences in the NiSP model with $\mu = 0$ are equal to the expectations under statistical independence, i.e.,

$$\bar{M}_k = C_k^n \bar{I}^k (1-\bar{I})^{n-k}, \tag{S55}$$

in which $\bar{I} = 1-\gamma/\beta$. In other words, the probability to be infected by k epidemiologically-interchangeable pathogens follows a binomial distribution with parameters n (the number of pathogens considered) and $p = \bar{I}$.

In the specific case $\mu = 0$, Eq. (S44) becomes

$$0=|\Gamma|\bar{F}\bar{J}_{\Omega_i}-\left((n-|\Gamma|)\bar{F}+|\Gamma|\gamma\right)\bar{J}_\Gamma+(n-|\Gamma|)\gamma\bar{J}_{\Lambda_i}, \tag{S56}$$

in which $\bar{F} = \beta - \gamma$. Eq. (S47) becomes

$$0=(n-k+1)\bar{F}\bar{M}_{k-1}-\left((n-k)\bar{F}+k\gamma\right)\bar{M}_k+(k+1)\gamma\bar{M}_{k+1}. \tag{S57}$$

Eq. (S55) implies

$$\bar{M}_{k-1} = \frac{C^n_{k-1}}{C^n_k}\frac{1-\bar{I}}{\bar{I}}\bar{M}_k = \frac{k}{n-k+1}\frac{1-\bar{I}}{\bar{I}}\,, \quad \text{and} \quad \bar{M}_{k+1} = \frac{C^n_{k+1}}{C^n_k}\frac{\bar{I}}{1-\bar{I}}\bar{M}_k = \frac{n-k}{k+1}\frac{\bar{I}}{1-\bar{I}}\bar{M}_k\,. \tag{S58}$$

Substituting the values in Eq. (S58) into the right side of Eq. (S57)

$$(n-k+1)\bar{F}\frac{k}{n-k+1}\frac{1-\bar{I}}{\bar{I}} - \big((n-k)\bar{F}+k\gamma\big) + (k+1)\gamma\frac{n-k}{k+1}\frac{\bar{I}}{1-\bar{I}} \tag{S59}$$

and simplifying leads to:

$$\bar{F}k\frac{1-\bar{I}}{\bar{I}} - (n-k)\bar{F} - k\gamma + \gamma(n-k)\frac{\bar{I}}{1-\bar{I}} = k\gamma - (n-k)\bar{F} - k\gamma + (n-k)\bar{F} = 0\,. \tag{S60}$$

Therefore, the values in Eq. (S55) are equilibrium values.

Similarly, in the specific case $\mu = 0$, Eq. (S48) becomes

$$0 = \bar{F}\bar{M}_{n-1} - (n\gamma)\,\bar{M}_n\,, \tag{S61}$$

and substituting the values in Eq. (S58) leads to

$$\bar{F}n\frac{1-\bar{I}}{\bar{I}} - n\gamma = n\gamma - n\gamma = 0\,. \tag{S62}$$

Lastly, in the specific case $\mu = 0$, Eq. (S50) becomes

$$0 = -\big(n\bar{F}\big)\bar{M}_0 + \gamma\bar{M}_1\,, \tag{S63}$$

and substituting the values in Eq. (S58) leads to

$$-n\bar{F} + \gamma n\frac{\bar{I}}{1-\bar{I}} = -n(\beta-\gamma) + n(\beta-\gamma) = 0\,. \tag{S64}$$

Hence, Eq. (S55) is the equilibrium solution of the NiSP model in the specific case $\mu = 0$.

4.6 Stochastic models

Continuous-time Markov chain. The continuous-time Markov chain model with pathogen-specific clearance rates has four additional events (Table 1).

Event number	Event	Rate	Change(s) to state variable(s) (ΔX)
8	Specific clearance of pathogen 1 from host singly-infected by pathogen 2	$\gamma_1 J_1 \Delta t + o(\Delta t)$	$J_1 \to J_1 - 1$<br>$J_\varnothing \to J_\varnothing + 1$
9	Specific clearance of pathogen 2 from host singly-infected by pathogen 1	$\gamma_2 J_2 \Delta t + o(\Delta t)$	$J_2 \to J_2 - 1$<br>$J_\varnothing \to J_\varnothing + 1$
10	Specific clearance of pathogen 1 from co-infected host	$\gamma_1 J_{1,2} \Delta t + o(\Delta t)$	$J_{1,2} \to J_{1,2} - 1$<br>$J_2 \to J_2 + 1$
11	Specific clearance of pathogen 2 from co-infected host	$\gamma_2 J_{1,2} \Delta t + o(\Delta t)$	$J_{1,2} \to J_{1,2} - 1$<br>$J_1 \to J_1 + 1$

Table 1: Additional transitions in the two-pathogen stochastic models.

Stochastic differential equations. Let $\mathrm{d}J = \tilde{f}\mathrm{d}t$ be the unscaled version of the deterministic model as specified in Eq. (S19-S20). The extension of matrix Σ in Eq. (24) of the main text, to include pathogen specific clearance is

$$\begin{bmatrix} \mu(N-J_\varnothing)+(F_1+F_2)J_\varnothing+\gamma_1 J_1+\gamma_2 J_2 & -F_1 J_\varnothing-(\mu+\gamma_1)J_1 & -F_2 J_\varnothing-(\mu+\gamma_2)J_2 & -\mu J_{1,2} \\ -F_1 J_\varnothing-(\mu+\gamma_1)J_1 & F_1 J_\varnothing+(F_2+\gamma_1+\mu)J_1+\gamma_2 J_{1,2} & 0 & -F_2 J_1-\gamma_2 J_{1,2} \\ -F_2 J_\varnothing-(\mu+\gamma_2)J_2 & 0 & F_2 J_\varnothing+(F_1+\gamma_2+\mu)J_2+\gamma_1 J_{1,2} & -F_1 J_2-\gamma_1 J_{1,2} \\ -\mu J_{1,2} & -F_2 J_1-\gamma_2 J_{1,2} & -F_1 J_2-\gamma_1 J_{1,2} & F_2 J_1+F_1 J_2+(\mu+\gamma_1+\gamma_2)J_{1,2} \end{bmatrix}, \tag{S65}$$

where $N - J_\varnothing = J_1 + J_2 + J_{1,2}$ and N is constant.

The new matrix G has dimension 4×11 due to the four additional events in Table 1 above, (see also Eq. (26) of the main text),

$$\begin{aligned} \mathrm{d}J_\varnothing &= \tilde{f}_0\mathrm{d}t-\sqrt{F_1 J_\varnothing}\,\mathrm{d}W_1-\sqrt{F_2 J_\varnothing}\,\mathrm{d}W_2+\sqrt{\mu J_1}\,\mathrm{d}W_5+\sqrt{\mu J_2}\,\mathrm{d}W_6+\sqrt{\mu J_{1,2}}\,\mathrm{d}W_7 \\ &\quad +\sqrt{\gamma_1 J_1}\,\mathrm{d}W_8+\sqrt{\gamma_2 J_2}\,\mathrm{d}W_9\,, \\ \mathrm{d}J_1 &= \tilde{f}_1\mathrm{d}t+\sqrt{F_1 J_\varnothing}\,\mathrm{d}W_1-\sqrt{F_2 J_1}\,\mathrm{d}W_4-\sqrt{\mu J_1}\,\mathrm{d}W_5-\sqrt{\gamma_1 J_1}\,\mathrm{d}W_8+\sqrt{\gamma_2 J_{1,2}}\,\mathrm{d}W_{11}\,, \\ \mathrm{d}J_2 &= \tilde{f}_2\mathrm{d}t+\sqrt{F_2 J_\varnothing}\,\mathrm{d}W_2-\sqrt{F_1 J_2}\,\mathrm{d}W_3-\sqrt{\mu J_2}\,\mathrm{d}W_6-\sqrt{\gamma_2 J_2}\,\mathrm{d}W_9+\sqrt{\gamma_1 J_{1,2}}\,\mathrm{d}W_{10}\,, \\ \mathrm{d}J_{1,2} &= \tilde{f}_{1,2}\mathrm{d}t+\sqrt{F_1 J_2}\,\mathrm{d}W_3+\sqrt{F_2 J_1}\,\mathrm{d}W_4-\sqrt{\mu J_{1,2}}\,\mathrm{d}W_7-\sqrt{\gamma_1 J_{1,2}}\,\mathrm{d}W_{10}-\sqrt{\gamma_2 J_{1,2}}\,\mathrm{d}W_{11}\,. \end{aligned} \tag{S66}$$

Covariance matrix at the endemic equilibrium. The new matrices A and B (Eq. (S4)) are

$$A = \begin{bmatrix} -\beta_1+\gamma_1+\mu & 0 \\ 0 & -\beta_2+\gamma_2+\mu \end{bmatrix} \tag{S67}$$

and

$$B = \begin{bmatrix} 2N(\gamma_1+\mu)\left(1-\dfrac{1}{R_{0,1}}\right) & \mu\bar{J}_{12} \\ \mu\bar{J}_{12} & 2N(\gamma_2+\mu)\left(1-\dfrac{1}{R_{0,2}}\right) \end{bmatrix}. \tag{S68}$$

The new steady-state covariance matrix $\bar{C}$ (Eq. (S8)) is

$$\bar{C} = \begin{bmatrix} \dfrac{1}{NR_{0,1}} & \dfrac{\mu\bar{J}_{1,2}}{N^2[\beta_1-(\gamma_1+\mu)+\beta_2-(\gamma_2+\mu)]} \\ \dfrac{\mu\bar{J}_{1,2}}{N^2[\beta_1-(\gamma_1+\mu)+\beta_2-(\gamma_2+\mu)]} & \dfrac{1}{NR_{0,2}} \end{bmatrix} \tag{S69}$$

where $\bar{J}_{12}$ is defined in Eq. (9) of the main text.

The covariance between pathogen 1 and pathogen 2 prevalences (the off-diagonal elements in Eq. (S69)) is

$$\begin{aligned} \bar{C}_{ij} &= \mathrm{cov}\left(\frac{I_1}{N},\frac{I_2}{N}\right) = \frac{\mu\bar{J}_{1,2}}{N^2[\beta_1-(\gamma_1+\mu)+\beta_2-(\gamma_2+\mu)]} & \text{(S70)} \\ &= \frac{(\beta_1+\beta_2)(\beta_1-\gamma_1-\mu)(\beta_2-\gamma_2-\mu)\mu}{N\beta_1\beta_2(\beta_1+\beta_2-\mu)(\beta_1-\gamma_1-\mu+\beta_2-\gamma_2-\mu)} \geq 0\,, & \text{(S71)} \end{aligned}$$

(for $i \neq j$) with equality if and only if $\mu = 0$ again (assuming $\beta_i > \gamma_i + \mu$, $i = 1,2$); in the latter case, the deviation from statistical independence is zero (Eqs. (4) and 5).

In the special case that $\beta_1 = \beta_2 = \beta$ and $\gamma_1 = \gamma_2 = \gamma$,

$$\frac{\partial\bar{C}_{ii}}{\partial\gamma} = -\frac{\mu}{\beta N(2\beta-\mu)} \leq 0\,, \tag{S72}$$

meaning that the positive covariance decreases as γ increases (unless $\mu = 0$), as expected.

5 The prevalence of co-infections can be equal to the product of the prevalences of interacting pathogens

We consider the same two-pathogen model as Eq. (S19), except we let $\mu = 0$. However, we include two interaction parameters $\sigma_1, \sigma_2 > -1$, such that the forces of infection of both pathogens are

$$F_1 = \beta_1(J_1 + (1+\sigma_1)J_{1,2}), \quad F_2 = \beta_2(J_2 + (1+\sigma_2)J_{1,2}). \tag{S73}$$

If $\sigma_i < 0$ (resp. > 0), then transmission of pathogen i from a co-infected host is lower (resp. greater) than from singly infected hosts ($i = 1, 2$). With these assumptions, the model is

$$\begin{aligned} \dot{J}_1 &= F_1 J_\varnothing - (F_2 + \gamma_1)J_1 + \gamma_2 J_{1,2}, \\ \dot{J}_2 &= F_2 J_\varnothing - (F_1 + \gamma_2)J_2 + \gamma_1 J_{1,2}, \\ \dot{J}_{1,2} &= F_2 J_1 + F_1 J_2 - (\gamma_1 + \gamma_2)J_{1,2}, \end{aligned} \tag{S74}$$

where $J_\varnothing = 1 - J_1 - J_2 - J_{1,2}$. If we let $I_1 = J_1 + J_{1,2}$ and $I_2 = J_2 + J_{1,2}$, then model (S74) is equivalent to

$$\begin{aligned} \dot{I}_1 &= \beta_1(I_1 + \sigma_1 J_{1,2})(1 - I_1) - \gamma_1 I_1, \\ \dot{I}_2 &= \beta_2(I_2 + \sigma_2 J_{1,2})(1 - I_2) - \gamma_2 I_2, \\ \dot{J}_{1,2} &= \beta_1(I_1 + \sigma_1 J_{1,2})(I_2 - J_{1,2}) + \beta_2(I_2 + \sigma_2 J_{1,2})(I_1 - J_{1,2}) - (\gamma_1 + \gamma_2)J_{1,2}. \end{aligned} \tag{S75}$$

Proceeding as in Section 1.1, let $P = I_1 I_2$ and $Z = P - J_{1,2}$. Thus,

$$\dot{Z} = -[\beta_1(I_1 + \sigma_1 J_{1,2}) + \beta_2(I_2 + \sigma_2 J_{1,2}) + \gamma_1 + \gamma_2]Z. \tag{S76}$$

Since the expression inside the brackets is positive, $Z(t) \to 0$ as $t \to \infty$. The prevalence of co-infection by interacting pathogens is asymptotically equal to the product of their prevalences. Therefore, $Z = 0$ does not imply pathogens do not interact.

6 Impact of environmental noise and transient behaviour

6.1 Environmental stochasticity

The stochastic model as presented in the main text allows only for demographic stochasticity, i.e. randomness caused by probabilistic effects in events such as infection or mortality. Temporal fluctuation in parameters controlling the events at which rates occur – i.e. environmental stochasticity – was therefore not included. It is natural to wonder whether our results hold when such an additional source of noise is included as a potentially confounding factor.

To understand whether and how environmental noise affects our conclusions, we considered an approach often used in biological models to assess the effect of environmental variability. In particular, we assumed each epidemiological parameter varies continuously over time and is temporally correlated with its past values, independently from other random variables. Each parameter (i.e. β_1, β_2 and μ) is modelled by a Stochastic Differential Equation (SDE) of the form

$$\mathrm{d}x(t) = r(\bar{x} - x(t))\,\mathrm{d}t + \sigma\sqrt{x(t)}\,\mathrm{d}W(t). \tag{S77}$$

This SDE is known as a Cox-Ingersoll-Ross (CIR) mean-reverting process (Allen, 2016; Iacus, 2008). This process has an asymptotic gamma distribution with mean and variance equal to $\bar{x}$ and $\sigma^2\bar{x}/(2r)$ (Allen, 2016; Iacus, 2008).

Mean-reverting processes more realistically model environmental variation in birth and death processes – such as our stochastic epidemic model – than linear white noise (Allen, 2016). Other mean-reverting processes have also been used to model environmental variability in biological populations (Marion et al., 2000; Varughese and Fatti, 2008), such as the Ornstein-Uhlenbeck (OU) mean-reverting process,

$$\mathrm{d}x(t) = r(\bar{x} - x(t))\,\mathrm{d}t + \sigma\,\mathrm{d}W(t)\,. \tag{S78}$$

However, the OU process has an asymptotic normal distribution with mean and variance equal to $\bar{x}$ and $\sigma^2/(2r)$ (Allen, 2016; Iacus, 2008). The advantage of the CIR process over the OU process – and the reason we use the CIR process here – is that sample paths are guaranteed to remain non-negative. The gamma distribution of epidemiological parameters is also more flexible than the normal distribution.

To check the effect of environmental noise on covariance, we modelled the three parameters (β_1, β_2, μ) using the SDE (S77), coupled with the SDE for the demographic variability presented in the main text (i.e. Eq. (26)). The mean value $\bar{x}$ for each parameter was set to the default (constant) value used in creating Fig. 2 in the main text. We used values of $r = 2$ and $\sigma = 0, 0.25, 0.5$ ($\sigma = 0$ has no environmental variability).

For all three levels of noise we considered, the relative deviation of the density of co-infected hosts from the density that would be expected if pathogen prevalences were independent (i.e. Λ) was reliably greater than zero (S2 Fig). Although the range of values that might be obtained as a point estimate of Λ from a single simulation was larger when there was more environmental noise, there was still a clear signal of a systematic deviation from statistical independence under all conditions we tested.

6.2 Transient behaviour

In applying our NiSP and NiDP models to data, we assume the host-pathogen interaction has equilibrated, and therefore use equilibrium values from the models to drive our statistical tests. This makes how quickly transient behaviour “washes out” of the system of potential interest. As indicated in the Discussion section of the main text, a full investigation is beyond our scope here. However, we repeated the randomisation analysis introduced in S1 Text Section 1.2 in a stochastic version of our model, to allow us to test how rapidly the difference between P and $J_{1,2}$ becomes apparent.

In particular, we fixed the pathogen parameters at the default values of $\beta_1 = 5, \beta_2 = 2.5$ and $\mu = 1$, and simulated the stochastic differential equation (with demographic noise) in a population of size $N = 1,000$. Choosing random initial conditions in the same manner as in S1 Text Section 1.2 (i.e. from a suitable Dirichlet distribution) and plotting out 95% intervals on the values of $\Lambda(t) = \frac{J_{1,2}(t) - P(t)}{P(t)}$, the relative deviation of the prevalence of co-infection from the product of the prevalences, as extracted at different times (S3 Fig) reveals that the initial conditions “wash out” of the system relatively rapidly. This remains the case both in the scenario in which all densities are chosen randomly and the scenario in which one pathogen is invading the other at equilibrium, although the difference takes longer to become apparent in the case in which one pathogen is invading. However, these results suggest it is reasonable to assume that transient behaviour is not overly influential in driving our results irrespective of the particular initial conditions that might be relevant in practice.

References

Allen, E. (2016). Environmental variability and mean-reverting processes. *Discrete & Continuous Dynamical Systems-Series B*, 21:2073–2089.

Iacus, S. M. (2008). *Simulation and inference for stochastic differential equations: with R examples*. Springer Science & Business Media.

Marion, G., Renshaw, E., and Gibson, G. (2000). Stochastic modelling of environmental variation for biological populations. *Theoretical Population Biology*, 57:197–217.

May, R. M. and Nowak, M. A. (1995). Coinfection and the evolution of parasite virulence. *Proceedings of the Royal Society, B*, 261:209–215.

O'Dea, E. B., Park, A. W., and Drake, J. M. (2018). Estimating the distance to an epidemic threshold. *Journal of the Royal Society: Interface*, 15:20180034.

Plemmons, R. J. (1977). M-matrix characterizations. I—nonsingular M-matrices. *Linear Algebra and its Applications*, 18:175–188.

Van Kampen, N. G. (1992). *Stochastic Processes in Physics and Chemistry*. Elsevier.

Varughese, M. M. and Fatti, L. (2008). Incorporating environmental stochasticity within a biological population model. *Theoretical Population Biology*, 74:115–129.
