# Supplementary material for: Coinfections by noninteracting pathogens are not independent and require new tests of interaction
Source: PLoS Biol. 2019 Dec 3;17(12):e3000551. doi: 10.1371/journal.pbio.3000551 (PMC6890165; doi:10.1371/journal.pbio.3000551)
Supplement: S2 Text — Gives more details on how data was selected and extracted, as well as discussing additional results of fitting the models that are not presented in the main text. (PDF) [file pbio.3000551.s002.pdf]

S2 Text. Sources of data and side results of model fitting

Organisation of this document

This document consists of three sections.

- 1. “Additional fitting of the NiSP model”.
  - Results of fitting the NiSP model to three additional data sets not considered in the main text. These studies do not concern a single pathogen species, and so the pragmatic assumption of epidemiological interchangeability between pathogens is less justifiable.
- 2. “Fitting the NiDP model”.
  - Re-tabulates the data sets on interactions between multiple *Plasmodium* spp. causing malaria reported by Howard et al. (2001), and describes how our criteria to allow a study to be considered led to us fixing on 41 particular studies – of the 73 reported – to analyse using our NiDP model.
- 3. “Fitting the models with specific clearance”.
  - Shows that fitting the NiSP model with specific clearance confirms qualitative results found from a model that does not include specific clearance as an additional parameter.

1 Additional fitting of the NiSP model

Results of fitting the NiSP model to data from four publications for strains of a single pathogen, that may plausibly be assumed epidemiologically-interchangeable (López-Villavicencio et al., 2007; Seabloom et al., 2009b; Chaturvedi et al., 2011; Koepfli et al., 2011), are presented in Fig. 3 of the main text. Results for three further data sets concerning different pathogens of a single host (Andersson et al., 2013; Moutailler et al., 2016; Nickbakhsh et al., 2016) are in S4 Fig.

For convenience the raw data as used in model fitting for these additional datasets are re-tabulated in S1 Table. Results of model fitting are summarized in S2 Table. Ambiguities needed to be resolved in collating these data from what is reported in the original publications. The data presented in Moutailler et al. (2016) are inconsistent, in as much as it is reported that a total of 267 ticks were tested, but the percentage data in the section “Co-infections and associations between pathogens” of the paper instead indicate 262 is the correct total. We have used the value 262 here. Misreporting of the number of uninfected hosts in reference Seabloom et al. (2009b) has been corrected by reference to the original data (Seabloom et al., 2009a) after personal communication with the authors.

2 Fitting the NiDP model

2.1 Sources of data

Howard et al. (2001) report results of analyzing 73 data sets concerning multiple *Plasmodium* spp. causing malaria (rows 68–140 of Table 1 in that paper). We re-analyzed the subset of these studies satisfying the additional constraints that they considered:

- interactions between three *Plasmodium* species (omits 16 rows corresponding to only two pathogens, *viz.* 73, 81, 86, 89-92, 104, 105, 107, 110, 120, 126, 134 and 139-140, as well as 2 rows corresponding to four pathogens, *viz.* 125 and 128);
- disease status of at least 100 individuals (omits 8 rows, *viz.* 72, 85, 87, 115, 129, 135, 136 and 138).

These constraints were imposed simply to reduce the number of studies, rather than because our methodology could not handle such data. We also omitted six of the remaining data sets – rows 83, 93, 94, 121, 122 and 131 – since we found it impossible to unambiguously reconcile the data as reported in the publication to counts of different types of infection. Most often this was because the data were reported as percentages rounded to a small number of significant figures, which did not unambiguously specify the raw number of individuals infected by each combination of pathogens. This left a final total of 41 data sets taken from 35 distinct papers: 24 data sets considering the three-way interaction between *P. falciparum*, *P. malariae* and *P. vivax* (denoted FMV in Howard et al. (2001)) and 17 data sets considering the three-way interaction between *P. falciparum*, *P. malariae* and *P. ovale* (denoted FMO in Howard et al. (2001)). The data sets are re-tabulated for convenience in S3 Table.

2.2 Recreating the analyses of Howard et al. (2001)

We did not explicitly recreate the analysis based on log-linear models as presented by Howard et al. (2001), since no information was given in the paper on how to handle sampling zeros (i.e. cases in which within an individual data set the count of individual infected by a particular combinations of pathogens is zero). Given the small size of many of the studies, this was quite common, affecting 34 of these 41 data sets.

The statistical difficulty is that at least one of the models involved in the model selection procedure cannot then reliably be estimated, since an estimated coefficient in a Poisson regression model tends to negative infinity. In turn this means that model selection based on log-likelihood ratio tests breaks down (Fienberg and Rinaldo, 2012). How such sampling zeros affect log-linear models with sparse data sets is an active area of current research in the methodological statistical literature, e.g. (Fienberg and Rinaldo, 2012).

It is unclear from what is presented in Howard et al. (2001) precisely how such cases were handled. Correspondence with the author of that paper that we were able to contact also did not reveal what precisely had been done in the original analysis. We note that, since our methods are based on multinomial sampling rather than Poisson counts, statistical difficulties surrounding sampling zeros simply do not affect our analyses.

3 Fitting the models with specific clearance

3.1 Fitting the models

All models were fitted after transformation to allow only biologically-meaningful values of parameters, by estimating $\log(\hat{\gamma}_i)$ to ensure only positive values of $\hat{\gamma}_i$ are permissible, and using these to estimate the infection rates after transformation, with $\log\big(\hat{\beta}_i/(\hat{\gamma}_i+1)-1\big)$ (which ensures $R_{0,i}>1$).

However, we noticed that this estimation method may return extremely high values of $\hat{\gamma}$ and $\hat{\beta}$ in the NiSP model. This is because we scaled β and γ relative to μ, while the optimal value of μ may be zero. The specific case $\mu = 0$ corresponds to statistical independence (see S1 Text Sections 4.3 and 4.4). When the data are close to being statistically independent, the estimation algorithm may diverge.

3.2 Results of fitting the two-parameter NiSP model

Results of model fitting are summarized in S4 Table; an example fit is shown in S5 Fig. Models were fitted by maximum likelihood, with model selection done via χ^2 tests on the likelihood-ratio (Bolker, 2008) or the Akaike Information Criterion (Sakamoto et al., 1986), depending on whether or not models were nested.

3.3 Fitting the NiDP model with specific clearance

Similarly to the NiSP model with specific clearance, estimated parameters occasionally diverge in the more complex version of the NiSP model with specific clearance, and very large numeric values of best-fitting epidemiological parameters can be obtained (but a reasonable value of R_0). Exploratory investigations suggested that fitting the NiDP model with specific clearance to the malaria data was affected by this type of identifiability issue, and so would therefore have required a specific treatment. Since our purpose here was not to draw conclusions about interactions among malaria species, but instead to show the utility of our overall approach, we did not pursue this analysis further.

References

Andersson, M., Scherman, K., and Råberg, L. (2013). Multiple-strain infections of *Borrelia afzelii*: a role for within-host interactions in the maintenance of antigenic diversity? *The American Naturalist*, 181:545–554.

Bolker, B. (2008). *Ecological Models and Data in R*. Princeton University Press, New Jersey.

Chaturvedi, A. K., Katki, H. A., Hildesheim, A., Rodríguez, A. C., Quint, W., Schiffman, M., Van Doorn, L.-J., Porras, C., Wacholder, S., Gonzalez, P., et al. (2011). Human papillomavirus infection with multiple types: pattern of coinfection and risk of cervical disease. *The Journal of Infectious Diseases*, 203:910–920.

Fienberg, S. E. and Rinaldo, A. (2012). Maximum likelihood estimation in log-linear models. *The Annals of Statistics*, 40:996–1023.

Howard, S., Donnelly, C., and Chan, M.-S. (2001). Methods for estimation of associations between multiple species parasite infections. *Parasitology*, 122:233–251.

Koepfli, C., Ross, A., Kiniboro, B., Smith, T. A., Zimmerman, P. A., Siba, P., Mueller, I., and Felger, I. (2011). Multiplicity and diversity of *Plasmodium vivax* infections in a highly endemic region in Papua New Guinea. *PLoS Neglected Tropical Diseases*, 12:e1424.

López-Villavicencio, M., Jonot, O., Coantic, A., Hood, M. E., Enjalbert, J., and Giraud, T. (2007). Multiple infections by the anther smut pathogen are frequent and involve related strains. *PLoS Pathogens*, 3:e176.

Moutailler, S., Valiente Moro, C., Vaumourin, E., Michelet, L., Tran, F. H., Devillers, E., Cosson, J.-F., Gasqui, P., Van, V. T., Mavingui, P., Vourc'h, G., and Vayssier-Taussat, M. (2016). Co-infection of ticks: the rule rather than the exception. *PLoS Neglected Tropical Diseases*, 10:1–17.

Nickbakhsh, S., Thorburn, F., Von Wissmann, B., McMenamin, J., Gunson, R., and Murcia, P. (2016). Extensive multiplex PCR diagnostics reveal new insights into the epidemiology of viral respiratory infections. *Epidemiology & Infection*, 144:2064–2076.

Sakamoto, Y., Ishiguro, M., and Kitagawa, G. (1986). *Akaike Information Criterion Statistics*. D. Reidel Publishing Company, Dordrecht.

Seabloom, E. W., Hosseini, P. R., Power, A. G., and Borer, E. T. (2009a). Data from: Diversity and composition of viral communities: coinfection of barley and cereal yellow dwarf viruses in California grasslands (doi:10.5061/dryad.2bj836).

Seabloom, E. W., Hosseini, P. R., Power, A. G., and Borer, E. T. (2009b). Diversity and composition of viral communities: coinfection of barley and cereal yellow dwarf viruses in California grasslands. *The American Naturalist*, 173:E79–E98.
