# Supplementary figures and images for: Coinfections by noninteracting pathogens are not independent and require new tests of interaction
Source: PLoS Biol. 2019 Dec 3;17(12):e3000551. doi: 10.1371/journal.pbio.3000551 (PMC6890165; doi:10.1371/journal.pbio.3000551)

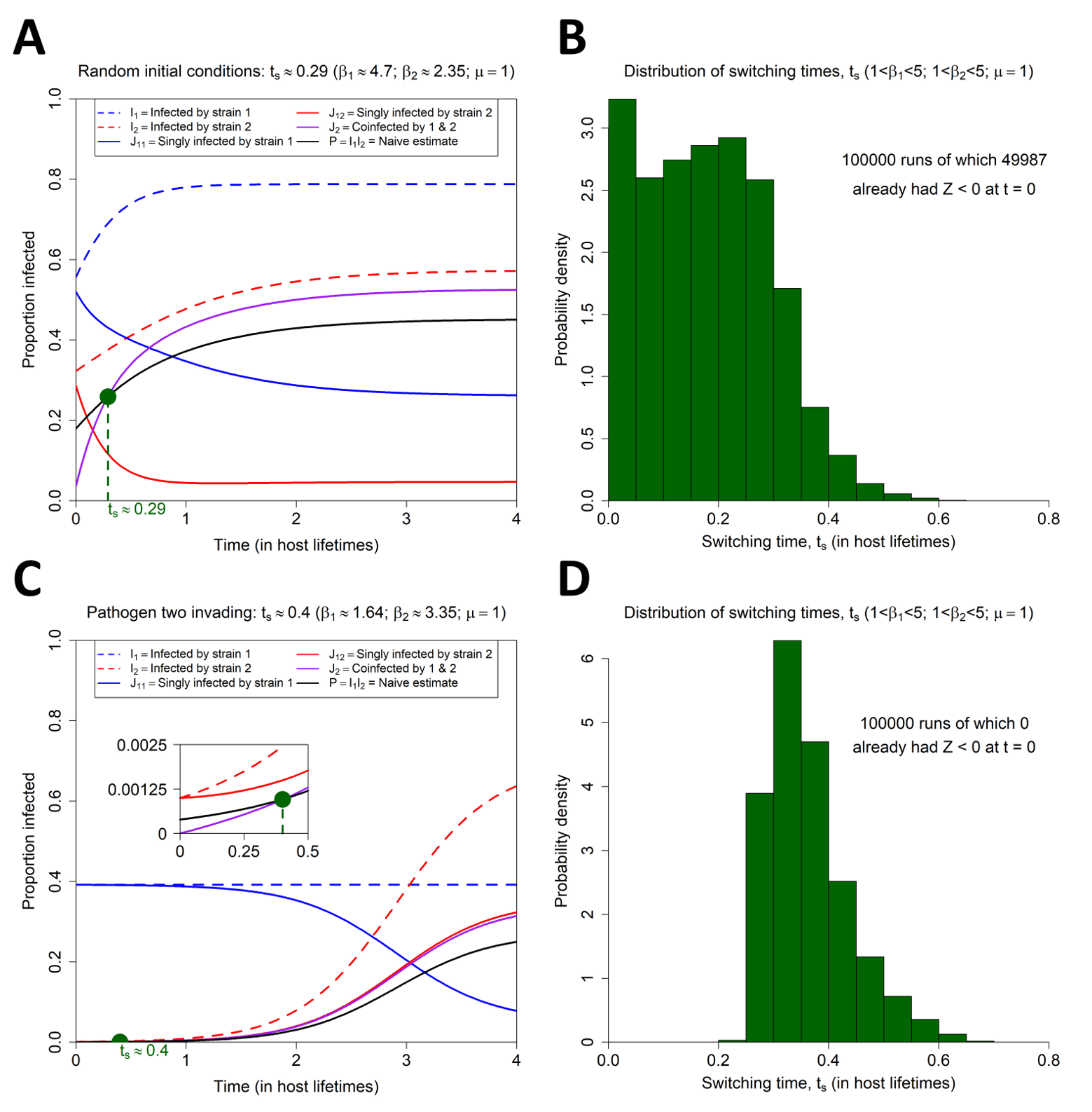

Supplement: S1 Fig — Panels (A) and (C) show how the switching time was calculated for both ‘random’ (A) and ‘one pathogen is invading’ (C) initial conditions (described in S1 Text Section 1.2) with epidemiological parameters chosen via a randomisation procedure (which ensured R0,1 and R0,2 were independently uniformly distributed between 1 and 5). The distribution of switching times over a large number of replicates (B and D) show the switching time is always less than the mean lifetime of an individual host for both initial condition scenarios. In both cases, any transient is therefore likely to have only limited impact (see also S1 Text Section 6). (TIF) [file pbio.3000551.s003.tif]

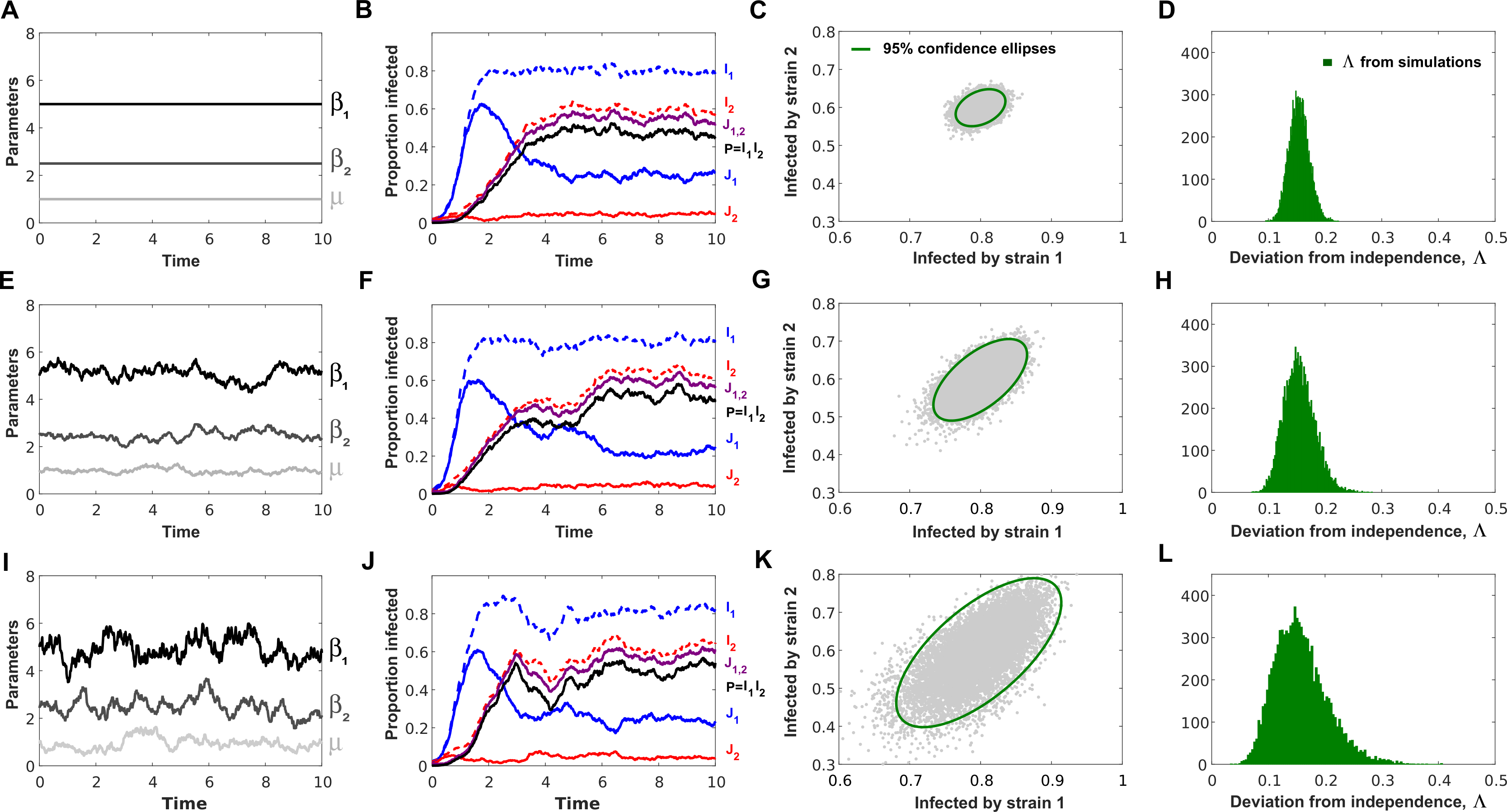

Supplement: S2 Fig — The stochastic differential equation version of the two-pathogen model was simulated 103 times, in a population N = 1,000, but the individual epidemiological parameters β1, β2, and μ were allowed to vary according to the Cox-Ingersoll-Ross process in Eq S77 in S1 Text (with mean values following the parameterisation used in Fig 2 of the main text). The three rows show results for σ = 0 (i.e., no environmental noise), σ = 0.25 (i.e., intermediate environmental noise), and σ = 0.5 (i.e., relatively high environmental noise). (A, E, and I) The evolution of the parameters over time in an individual replicate simulation. (B, F, and J) The corresponding trajectories for the density of infected hosts. (C, G, and K) The distribution of 103 point estimates of (I1,I2) when T = 10. (D, H, and L) The empirical distribution of the relative deviation from statistical independence Λ=(J¯1,2−P¯)/P¯ over the 103 simulations at each level of noise. For all three levels of noise, the full distributions of Λ remain reliably above zero. (Note that since the level of noise is set to zero for the results shown in the top row, panels B, C, and D essentially replicate Fig 2B, 2C and 2D in the main text). (TIFF) [file pbio.3000551.s004.tiff]

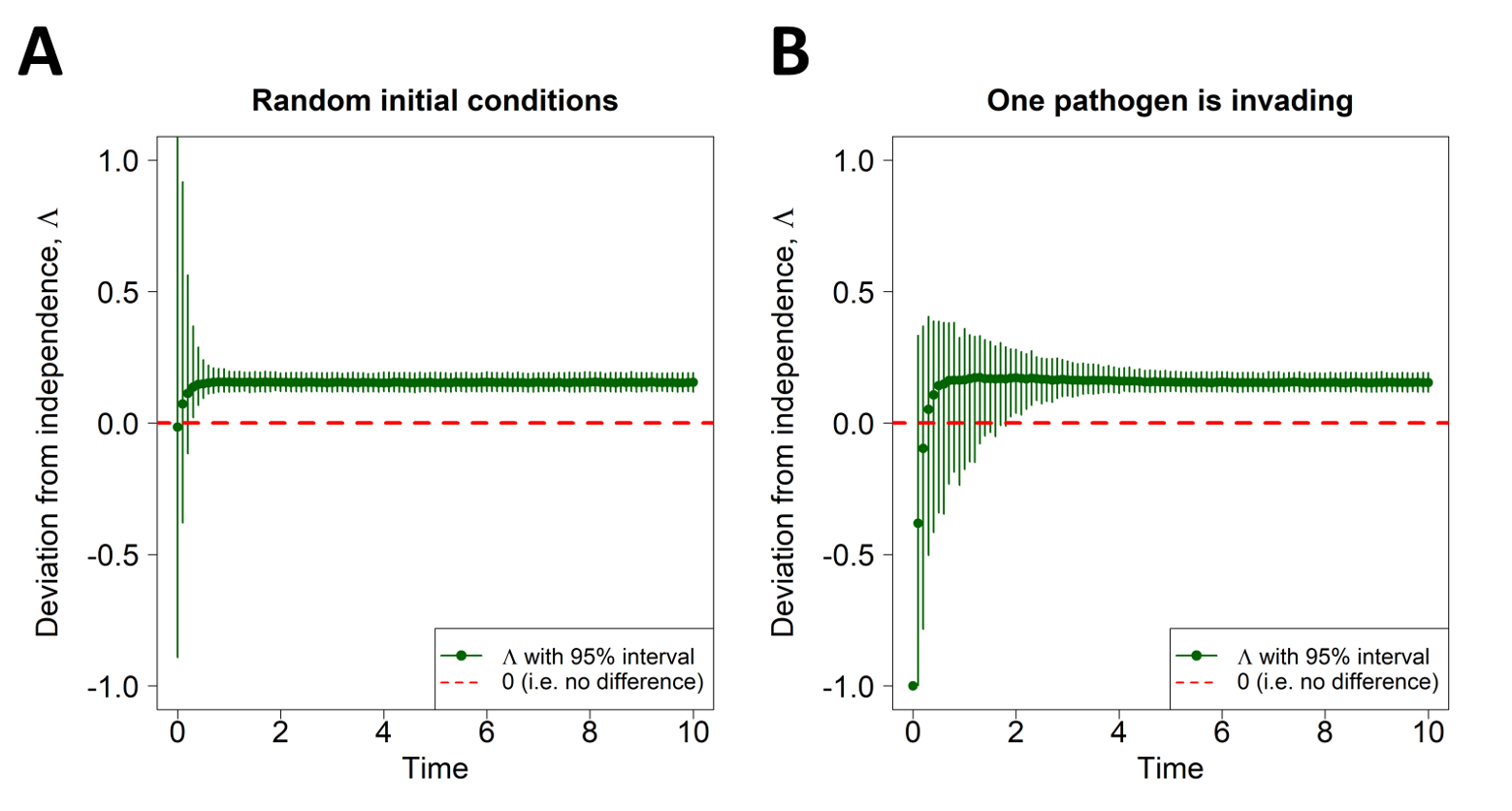

Supplement: S3 Fig — The stochastic differential equation version of the two-pathogen model with the parameterisation used in Fig 2 of the main text was simulated 1,000 times with random initial conditions, in a population N = 1,000. The 95% interval on the value of Λ as extracted from individual simulations at different times is shown for different assumptions on the initial conditions (see also S1 Text, Section 1.2). (A) Random initial conditions, with densities of all four state variables chosen at random. (B) One pathogen is invading the other, which is initially at equilibrium. (TIF) [file pbio.3000551.s005.tif]

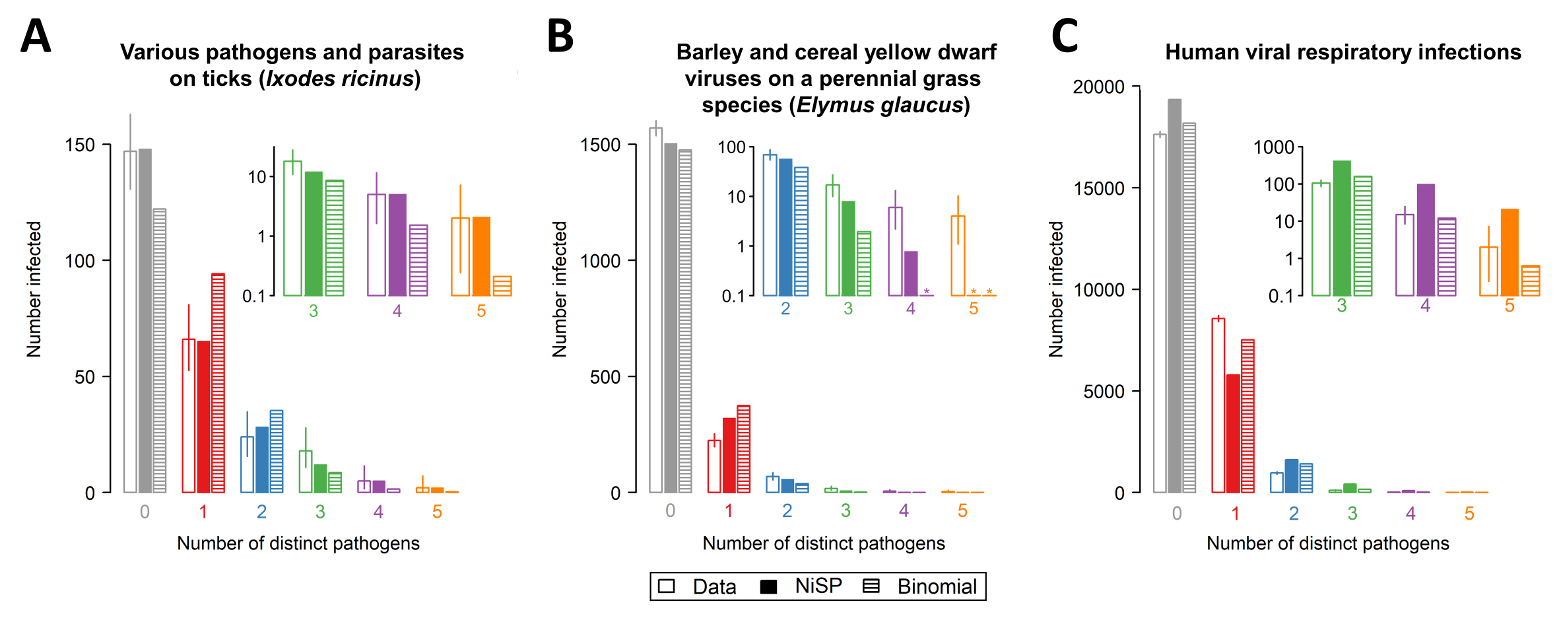

Supplement: S4 Fig — Model-fitting results are shown for (A) pathogens of Ixodes ricinus ticks [50], (B) barley and cereal yellow dwarf viruses [49], and (C) human respiratory viruses [48]. Insets to each panel show a ‘zoomed-in’ section of the graph corresponding to high multiplicities of pathogen coinfection, using a logarithmic scale on the y-axis for clarity. Asterisks indicate predicted counts smaller than 0.1. For the data shown in (A), there is no evidence that the NiSP model does not fit the data, and so our test indicates the pathogens do not interact. For the data shown in (B), although the NiSP model is a better fit to the data than the binomial model, there is evidence of lack of goodness of fit, and so our test indicates these pathogens interact (or are epidemiologically different). For the data shown in (C), although the binomial model is a better fit to the data than the NiSP model, there is evidence of lack of goodness of fit, and again it can be concluded that these pathogens interact (or are epidemiologically different). The underlying data for this figure can be found in S9 Data, S10 Data, and S11 Data. NiSP, Noninteracting Similar Pathogens. (TIF) [file pbio.3000551.s006.tif]

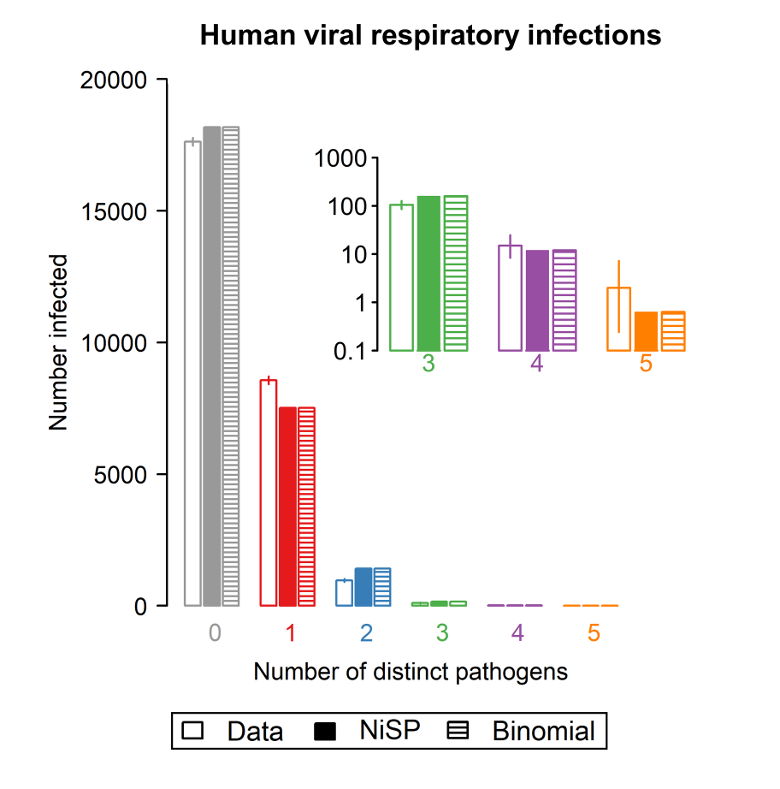

Supplement: S5 Fig — Model-fitting results are shown for human respiratory viruses [48]. The inset shows a ‘zoomed-in’ section of the graph corresponding to high multiplicities of pathogen coinfection, using a logarithmic scale on the y-axis for clarity. The best-fitting NiSP model converges to the binomial model in this case (which is a special case of NiSP for μ = 0, see S1 Text [Section 4]). The underlying data for this figure can be found in S12 Data. NiSP, Noninteracting Similar Pathogens. (TIF) [file pbio.3000551.s007.tif]
