# Supplementary material for: Coinfections by noninteracting pathogens are not independent and require new tests of interaction
Source: PLoS Biol. 2019 Dec 3;17(12):e3000551. doi: 10.1371/journal.pbio.3000551 (PMC6890165; doi:10.1371/journal.pbio.3000551)
Supplement: S1 Table — The data sets include pathogens of I. ricinus ticks [50], barley yellow dwarf viruses [49], and respiratory viruses [48]. The underlying data for this table can be found in S1 Data. NiSP, Noninteracting Similar Pathogens. (PDF) [file pbio.3000551.s008.pdf]

S1 Table

Pathogens with *n* distinct types, strains or clones	n	Observed counts, O_k										Total
		0	1	2	3	4	5	6	7	8	9	N
Pathogens of *Ixodes ricinus* ticks	37	147	66	24	18	5	2	-	-	-	-	262
Barley and cereal yellow dwarf viruses	5	1570	224	69	17	6	4	-	-	-	-	1890
Respiratory viruses	11	17630	8568	964	105	15	2	-	-	-	-	27284
