# Supplementary material for: Coinfections by noninteracting pathogens are not independent and require new tests of interaction
Source: PLoS Biol. 2019 Dec 3;17(12):e3000551. doi: 10.1371/journal.pbio.3000551 (PMC6890165; doi:10.1371/journal.pbio.3000551)
Supplement: S2 Table — The NiSP model was highly supported over the binomial model (ΔAIC≫10) in all cases tested but one (respiratory viruses), in which the binomial model is highly supported over the NiSP model. The final column of the table corresponds to the GoF test of the NiSP model; values p>0.05 correspond to lack of evidence for failure to fit the data, and so the NiSP model is adequate for the data concerning pathogens of I. ricinus ticks [50]. AIC, Akaike information criterion; GoF, goodness of fit; NiSP, Noninteracting Similar Pathogens. (PDF) [file pbio.3000551.s009.pdf]

S2 Table

	NiSP		Binomial			GoF
	R_0	*L*	*p*	*L*	ΔAIC=2ΔL	*p*
Pathogens of *Ixodes ricinus* ticks	1.021	-314.1	0.020	-329.3	30.5	**0.476**
Barley and cereal yellow dwarf viruses	1.051	-1180.8	0.048	-1261.9	162.2	0.000
Respiratory viruses	1.037	-22619.0	0.036	-21731.9	-1774.2	0.000
