# Supplementary material for: Coinfections by noninteracting pathogens are not independent and require new tests of interaction
Source: PLoS Biol. 2019 Dec 3;17(12):e3000551. doi: 10.1371/journal.pbio.3000551 (PMC6890165; doi:10.1371/journal.pbio.3000551)
Supplement: S3 Table — The asterisks indicate that the corresponding data sets were extracted from the large compendium collated in 1930 by Knowles and White. The number in the leftmost column shows the number of the relevant row in Table 1 of [17]. The rows with (!) correspond to studies for which the total number of individuals sampled as reported by [17] do not match what we found on interrogating the original paper; in all cases, we used the corrected values as shown in the table. The notation ‘X’ (in FX, MX, or FMX) corresponds either to ‘V’ (i.e., P. vivax, upper part of the table, data sets 74–137) or to ‘O’ (i.e., P. ovale, lower part of the table, data sets 68–103). The underlying data for this table can be found in S2 Data. (PDF) [file pbio.3000551.s010.pdf]

S3 Table

			Observed counts, O_Γ							
		N	Ø	F	M	V	FM	FX	MX	FMX
74	Léger et al. (1923)	250	83	111	49	1	6	0	0	0
75	Bédier et al. (1924)	135	45	58	27	3	2	0	0	0
76	Knowles and White (1930)	809	642	149	12	1	2	2	0	1
82 (!)	Dorolle (1927)	652	232	258	64	54	32	12	0	0
84	Phillips (1923)*	645	409	112	10	109	0	4	1	0
88	Lalor (1913)*	207	94	47	21	40	0	3	2	0
106	Wilson (1936)	3393	1784	1103	87	19	244	63	2	91
108 (!)	Borel and Levanan (1927)	1249	885	227	23	92	3	16	3	0
109 (!)	Borel and Levanan (1927)	1022	947	19	12	39	0	4	1	0
111	Banerjea (1930)*	1519	578	225	7	668	0	41	0	0
112	Khambata (1913)*	112	72	26	8	4	0	1	1	0
113	Ramsay (1928)*	1514	1073	268	9	160	0	3	1	0
114	Bailey (1928)*	1068	547	396	67	35	18	5	0	0
116	Masterman (1913)	700	238	317	75	55	4	9	2	0
117	Angus (1919)*	40168	28936	2614	9	8483	0	126	0	0
118	Gordon et al. (1991)	268	208	14	6	30	1	6	3	0
119	Lalor (1912)*	151	52	38	13	33	4	6	4	1
123	Carter (1927)*	11260	9510	170	568	986	2	4	19	1
124	Schnuffer (1938)	3266	2148	593	234	196	42	30	15	8
127	Banchongaksorn et al. (1996)	913	487	221	5	179	0	21	0	0
130	Treadgold (1918)	540	396	3	1	136	0	4	0	0
132	Collins et al. (1988)	614	407	151	11	19	3	21	2	0
133	Mizushima et al. (1994)	506	231	144	0	81	1	39	4	6
137	United Fruit Co. (1925)*	2742	1973	435	14	299	0	21	0	0
68	Campbell et al. (1987)	147	77	56	0	1	11	1	0	1
69	Campbell et al. (1987)	142	26	68	3	0	40	4	0	1
70	Campbell et al. (1987)	196	41	112	2	0	25	10	0	6
71	May et al. (1997)	230	40	123	1	0	32	7	0	27
77	Alifrangis et al. (1999)	126	5	72	2	0	31	13	0	3
78	Hellgren et al. (1994)	163	32	105	5	0	20	1	0	0
79	Thomson et al. (1994)	1465	770	641	17	2	30	5	0	0
80	Gbary et al. (1988)	735	444	234	22	7	20	7	1	0
95	Deloron et al. (1989)	1465	770	641	17	2	30	5	0	0
96	Deloron et al. (1989)	245	130	95	0	0	16	4	0	0
97	Deloron et al. (1989)	253	126	109	4	0	13	0	0	1
98	Deloron et al. (1989)	225	136	82	0	0	3	4	0	0
99	May et al. (1997)	159	97	56	0	1	3	2	0	0
100	Trape et al. (1992)	2465	2372	85	6	0	1	1	0	0
101	Trape et al. (1994)	8539	2208	4254	133	50	1435	227	3	229
102	Molineaux et al. (1980)	7026	2658	3295	143	36	742	108	6	38
103	Molineaux et al. (1980)	6526	3474	2015	183	15	757	42	2	38
