# Supplementary material for: Coinfections by noninteracting pathogens are not independent and require new tests of interaction
Source: PLoS Biol. 2019 Dec 3;17(12):e3000551. doi: 10.1371/journal.pbio.3000551 (PMC6890165; doi:10.1371/journal.pbio.3000551)
Supplement: S4 Table — Parameters for the best-fitting variant of the NiSP model for each pathogen species, strain, or clone are highlighted in bold; the two-parameter model is supported in cases for which p<0.05 in the ‘Model Selection’ part of the table (including human papillomavirus and malaria [P. vivax]). The NiSP model was highly supported over the binomial model (ΔAIC≫10) in all cases tested but one (human respiratory viruses). The final column of the table corresponds to the GoF test of the best-fitting model; values p>0.05 correspond to lack of evidence for failure to fit the data, and so the NiSP model is adequate for the data concerning human papillomavirus and pathogens of I. ricinus ticks. These results are qualitatively identical to those for the model without specific clearance as presented in the main text. Note that in the NiSP model, β and γ are scaled relative to μ. This is why β and γ of NiDP reach extremely high values for respiratory viruses. Parameter estimation tends to μ = 0, which actually corresponds to the binomial model, which has one fewer parameter (see S1 Text Section 4.5 and S5 Fig). Hence, ΔAIC = −2 for respiratory viruses, since the NiSP model requires one additional parameter compared to the binomial model. AIC, Akaike information criterion; GoF, goodness of fit; NiDP, Noninteracting Distinct Pathogens; NiSP, Noninteracting Similar Pathogens. (PDF) [file pbio.3000551.s011.pdf]

S4 Table

	NiSP (β only)		NiSP (β & γ)			Model selection		Binomial			GoF
	β	L	β	γ	L	χ^2	p	p	L	ΔAIC	p
Human papillomavirus	1.032	-6580.9	**1.178**	**0.142**	-6573.1	7.794	**0.005**	0.031	-6868.8	589.3	**0.986**
Pathogens of *I. ricinus* ticks	**1.021**	-314.1	1.161	0.137	-313.7	0.360	0.549	0.020	-329.3	30.5	**0.476**
Anther smut (*M. violaceum*)	**1.009**	-611.4	1.009	0.000	-611.4	0.000	1.000	0.009	-690.8	158.8	0.000
Barley yellow dwarf viruses	**1.051**	-1180.8	1.051	0.000	-1180.8	0.000	1.000	0.048	-1261.9	162.2	0.000
Borrelia afzelii on bank voles	**1.044**	-652.1	1.044	0.000	-652.1	0.000	1.000	0.040	-799.0	293.8	0.000
Malaria (*Plasmodium vivax*)	1.021	-3169.2	**1.162**	**0.138**	-3164.6	4.588	**0.032**	0.021	-3467.3	603.5	0.000
Respiratory viruses	1.037	-22619.0	4.7×10^9	4.6×10^9	-21731.9	887.057	0.000	**0.036**	-21731.9	-2.0	0.000
