# Supplementary material for: Coinfections by noninteracting pathogens are not independent and require new tests of interaction
Source: PLoS Biol. 2019 Dec 3;17(12):e3000551. doi: 10.1371/journal.pbio.3000551 (PMC6890165; doi:10.1371/journal.pbio.3000551)
Supplement: Attachment — Submitted filename: PLOS_Biology_Response_To_Reviews.pdf [file pbio.3000551.s024.pdf]

Reviewer #1:

This manuscript tackles an issue of growing recognition - how to infer the presence of interspecific parasite interactions from cross-sectional data. This is a problem since, particularly for wildlife systems, longitudinal or experimental data are rare, so we often have to rely on data of this form to draw conclusions of how and to what extent co-infecting parasites interact with each other. This ms very neatly demonstrates that the fundamental assumption that non-interacting parasites should show statistical independence in their co-occurrence is wrong, due to the likely positive correlations that arise due to the confounding effect of host age. The authors then show how this non statistical independence can be accounted for, nicely illustrated with re-fitting models to previously published data.

Overall this is a very well written, elegant and informative ms, and I think it very clearly makes important points about some of our fundamental assumptions about our null models regarding patterns of co-association between non-interacting species. I only have a couple of very minor additional suggestions:

> ***Response.*** *We thank Reviewer #1 for their careful reading of our m/s and their very positive assessment of our work.*
>
> ***Action.*** *None requested. We respond inline to each point made below.*

- Equation 4 [now Equation 5] is presented as the measure of deviation of the prevalence of co-infection from that of statistical independence, and how this is affected by host longevity - that's fine, but I found Equation 9 [now Equation 4] (in the Methods) to be a much nicer/clearer representation, clearly showing that if mu=0 (essentially immortal hosts) then the observed co-infection prevalence equals the expected proportion assuming independence - whereas as mu increases (mean host lifespan decreases) this difference accentuates. I wonder if Eq 9 [now Equation 4] can be brought into the main text to help reinforce these points.

> ***Response.*** *While the middle part of what used to be Eq. (4) and is now Eq. (5) has the death rate* μ *as its sole numerator, and so already highlights the first aspect of the behaviour identified by Reviewer #1 (i.e. what happens when* μ*=0), we agree that what used to be Eq. (9) makes the behaviour as* μ *increases away from zero clearer.*
>
> *We have therefore moved Eq. (9) from the Methods to the Results.*
>
> ***Action.*** *Added text and equation to the Results section (L163-167; Eq. (4)), with corresponding deletion from the Methods (L455 is the text introduced into the Methods in making this update). Some references to numbered equations in the main text that are made in the Supplementary Information were affected by this reordering: this has led to a number of (very small) changes, restricted entirely to numbering of equations (e.g. L204 in Supplementary Information, although there is a large number of similar changes).*

- Line 153 states the "deviation [between co-infection prevalence and that required by statistical independence] is zero if an only if the host natural death rate = 0". Yes, that's strictly true, but (from Eqns 4/9 [now Equations 4 and 5]] it would also be approximately true if the R0 and/or transmission rates of either or both pathogens are large - the deviation tends to zero as beta1 and/or beta2 increase. Biologically this means we would expect to see the assumption of statistical independence violated for pathogens with high transmission rates/R0s, whereas those with very low transmission rates (assuming

R0 remains > 1) we would likely not see much departure from the assumption of statistical independence. It might be useful to make this point explicitly here.

> ***Response.*** *As Reviewer #1 states, as the value of R_0 for either pathogen increases, the relative deviation from statistical independence decreases. This means that if one or both pathogens have a very large value of R_0 the deviation is approximately zero.*
>
> *However, the implication is actually in the other direction to what is stated by Reviewer #1. If either R_0 is very large then there would not be a large departure from what would be expected under the assumption of statistical independence. It is only when neither R_0 is large that the deviation will be of a reasonable size. This is because if R_0 for – say – pathogen 1 is very large then almost all hosts infected with pathogen 2 will become co-infected in our model (or* vice versa*). Therefore both our new model and the assumption of statistical independence would lead to virtually identical results under these conditions.*
>
> *We agree this is an interesting point, and have added some text to explicitly note it.*
>
> ***Action.*** *Added text to Results (L172-177).*

- I found it very interesting that this ms highlights the expectation of positive correlations among parasites, even if they don't interact (or presumably even if they negatively interact, providing the confounding age effect is sufficiently strong) - as this matches the finding in Fenton et al 2014 IJP that most correlation-based cross-sectional analyses revealed positive associations between pairs of parasites that are known to interact negatively with each other (their Fig 2). I wonder if it's useful to make this point in the Discussion somewhere, as a theoretical explanation for this empirically-observed result.

> ***Response.*** *Thank you for highlighting this interesting reference. We have added a new paragraph to the discussion, referencing this as well as a somewhat similar paper by Dallas* et al. *(2019). However, we are careful not to over-speculate given both of these studies focus on parasites, for which the S-I-S model is not the typical epidemiological modelling paradigm.*
>
> ***Action.*** *Added text to Discussion (L296-312).*

Reviewer #2:

This paper uses SIS models to show why systems of non-interacting pathogens will—counter to intuition—always appear to exhibit statistical dependence according to conventional metrics, simply on account of positive correlations between I1 and I2 that are generated when coinfected hosts die. The authors go on to develop some statistical tests based on this insight that can account for this effect, and they re-analyze a number of datasets with the new tests. The problem is tackled quite thoroughly by the authors, who present several lines of methodological evidence along with numerical simulations and empirical analyses. I think this paper will be of broad interest to the infectious disease modelling community, and even more broadly in population biology and epidemiology. The paper is also very well-written and the figures are clear. I only have a few minor revisions to suggest:

> ***Response.*** *We thank Reviewer #2 for their positive assessment of our work and complementary comments on the quality of our exposition.*

Action. *None requested. We respond inline to each point made below.*

1. The analysis relies heavily on the SIS natural history which is suitable for chronic infections (as the authors point out). However, they apply their test to HPV, which exhibits short-lived natural immunity followed by clearance, in most cases. In the Discussion section, the authors should discuss in more detail how other natural histories would influence their findings, including their finding of independence for HPV strains. I assume it would make their findings conservative.

Response. *We emphasized in the original submission (e.g. text on L340-345 of the revised submission, retained unchanged from the original text) that we were using HPV as a convenient case study for which data were readily available. We certainly agree that there are particular details of HPV epidemiology that are not captured by such a simple model.*

However, we have now included additional text on how our extension to the model to allow for pathogen clearance within the lifetime of an individual host does <u>not</u> *challenge the key result (by explicitly highlighting how results of testing precisely this are presented in the Supplementary Information, as they were in the original submission).*

Action. *Added further text on this point to the Discussion (L333-340).*

2. The proof in S1.1 only shows that Z becomes negative after a finite time t1, meaning that it could be positive before that. Hence, depending on how large t1 is, we could observe either J1,2 > I1*I2 or vice versa. Please comment and explain whether this is a significant limitation, and adjust writing in main text as needed.

Response. *Thank you for raising an interesting point.*

While it appears rather difficult to say very much about the general behaviour of the "switching time" in our deterministic two pathogen model by way of mathematical analysis, this can be studied numerically. We therefore performed a numerical investigation in the two-pathogen model, characterizing the time at which Z – i.e. the difference between $J_{1,2}$ and I_1 I_2 – first becomes negative. We attempted to investigate the full range of behaviours possible in our two-pathogen model by randomly choosing the values of R_0 for each pathogen within certain bounds.

We focused on two scenarios for initial conditions – either that nothing is assumed a priori*, with all initial densities chosen at random, or that one pathogen is invading the other, and so that there is a small (random) initial density of one pathogen invading the other when it is initially at equilibrium. In both cases, for all parameters we tested, the switching time is relatively short, and the switch from positive to negative values of Z occurs within a single host lifetime. So while it can take a little time for the prevalence of co-infection to first exceed the product of prevalence, in relation to host lifetimes at least, this transient behaviour is very unlikely to be significant.*

Action. *These results are presented in the (entirely new) Supplementary Information S1.1.2 and Supplementary Figure S1 (altered text is on L16-17; L64; L80-119; L124-127 of the Supplementary Information). We have adjusted the main text (Results: L144-148; Discussion: L424-429) to reference this additional analysis.*

3. Following on comment #2, the authors could either expand their numerical analysis depicted in Figure 2 to explore whether transient dynamics have any interesting impact on the relationship between J1,2 and I1*I2. Alternatively, they could include some speculation about the impact of transients in the Discussion section.

> ***Response.*** *As described above, we now present a new Supplementary Figure S1 exploring the switching time. Of course, the switching time alone does not really tell us very much about when a signal will become detectable from real data, since that depends on the extent of the difference between the density of co-infection and the product of the prevalences, rather than whether or not one is larger than the other. We therefore also performed an initial analysis for our default parameter set of how quickly a signal would be likely to be detectable, by repeatedly simulating the stochastic differential equation version of our two-pathogen model, again with randomized initial conditions.*
>
> *These results are presented as part of the new Supplementary Information S1.6.2 (see also response to Reviewer #3, below), which – by way of numerical experiments testing as a function of time that has passed since* t = *0 the size of the relative difference between the density of co-infecteds and the product of prevalences – show that the difference is likely to rapidly become visible, irrespective of the particular scenario on initial conditions that is chosen. These results are presented in the new Supplementary Figure S3.*
>
> *It would of course be possible to go beyond this, and in particular to use simulated data to more systematically explore the timing of the first time at which an interaction becomes detectable as a function of: i) size of the data set that is being tested in our NiDP or NiSP model; ii) the parameter values for each of the pathogens (i.e. the values of $R_{0,1}$ and $R_{0,2}$); iii) the relative size of the deviation from equilibrium at the time of testing (i.e. the initial conditions); iv) the effect size of interaction that the test must be able to detect. However, doing this would only give us a firmer understanding of the case in which there are only two pathogens (and in which the data set was simulated)!*
>
> *Given this, we prefer to take the other option – thankfully explicitly suggested by Reviewer #2! – i.e. to simply augment the additional numerical work that was described above with some coverage of the likely importance of transients in the discussion.*
>
> ***Action.*** *We have added new text to the Results section (L158-161) briefly pointing the interested reader to these new analyses, with more extensive coverage in the Discussion (L416-423; L429-437). The Supplementary Information has been altered with the new Section S1.6.2 (L387-408) and addition of Supplementary Figure S3.*

Reviewer #3:

It was a pleasure to read the manuscript by Hamelin et al. touching on the important question in ecology and epidemiology whether co-infections of non-interacting pathogens are statistically independent. Building upon prior work by Kucharski et al., the manuscript showcases a wide range of tools from ODE and SDE modelling to statistics. It provides a practical tool to test for interactions between pathogens based on cross-sectional data for chronic infectious diseases explicitly accounting for non-independence of co-infection prevalences. The availability of source code and data gathered from previous studies will further benefit the research community.

Response. *We thank Reviewer #3 for their extremely positive assessment of our work and complementary comments on its potential utility for the community.*

Action. *None requested. We respond to the particular points made below.*

Major comments

In the SDE model, especially Figure 2B, the authors show only a single replicate to underpin non-independence, i.e. that the product prevalence is lower the the co-infection prevalence. It would be interesting to see a more representative number of replicates (with mean and confidence interval bands). Would then the product prevalence still be statistically significantly different from the the co-infection prevalence?

Response. *Thank you for making an important point, which we agree would be of interest to many readers. We have therefore made two changes to Figure 2 in the main text, which illustrate that our result generalizes beyond the single replicate we originally chose (note: we of course chose that replicate entirely at random!).*

We now show the variability in point estimates of I1 and I2 from 1,000 replicates of our model in Figure 2C. This has the pleasing side-effect of very clearly showing that the analytical approximation (ellipses in Figure 2C) is a very good match to the behaviour of the full stochastic model over many replicates (e.g. 11 of the 1000 grey dots are outside the range of the 99% confidence ellipse: 10 "should" be outside if the prediction were "perfect").

We have also added a new Figure 2D, which explicitly shows the way in which – for the default parameterization of our model – the difference between the product of the prevalances and the density of co-infected hosts is very reliably distinguishable from 0 over many runs of our model.

Action. *Updated Figure 2 (Results in the main text) to include additional information as part of panel C, as well as an entirely new panel D, with associated changes to the figure caption. There is also some new text in the Results highlighting these changes (L153-155)*

Concerning the positive correlation between pathogen prevalences resulting from the covariance matrix of fluctuations near the equilibrium, the authors mentioned the works of O'Dea et al. (2018). In the cited paper, assumptions on environmental (multiplicative gamma white) noise play an import role in the calculation of the covariance matrix. Would you expect that the absence of biological interactions between pathogens (as seen in the HPV example) would still be identifiable with your approach if your model was augmented by such environmental factors?

Response. *Again, thank you for this comment.*

As Reviewer #3 has identified, the stochastic model as presented in our original submission allows only for demographic stochasticity, i.e. randomness caused by probabilistic effects in events such as infection or mortality. Temporal fluctuation in parameters controlling the events at which rates occur – i.e. environmental stochasticity – was therefore not included. It is natural to wonder whether our results hold when such an additional source of noise is included.

Reviewer #3 will be reassured to hear that our results generalize.

We checked this by extending our model to allow the epidemiological parameters β_1, β_2 and μ to vary according to a mean-reverting Cox-Ingersoll-Ross process. This makes the individual parameter values follow a Gamma distribution (of specified variance, which in this context corresponds to the level of environmental noise). Demographic variability is still included.

Even with this additional source of variation, there is still a clear signal of a systematic deviation from statistical independence. Large environmental variances make the range of values that might be obtained as a point estimate of the relative deviation wider. However, we note that the deviation was reliably above zero for all simulations and levels of environmental noise that we tested (including a parameterization under which the level of noise was relatively large). It therefore seems plausible that the deviation would remain detectable in practice, under a wide range of conditions.

Action. *Added new section to the Supplementary Information S1.6.1, with associated Supplementary Figure S2 (the changes are to L345-386 of the Supplementary Information). This new material is referenced on L155-158 in the Results section of the m/s.*

Similarly, in your discussion section you touch upon whether structural model constraints (e.g. age classes) should necessarily be accounted for in order to test for non-interaction between pathogens. It would be interesting to sketch strategies how to deal with structures (e.g. networks, meta-populations, immunity) that are relevant for chronic diseases and that might mask interactions.

Response. *Thank you for this comment: we agree this idea is worth discussing in the main text. We have added a new paragraph to the discussion sketching out how additional sources of heterogeneity might be included. However, in order to not overstate our case, we also acknowledge how more data – taken from multiple time points – would then perhaps be required to unambiguously characterize interactions.*

Action. *New paragraph in Discussion (L392-414)*

Minor comments

Main text
line 343 replace “of of” by “of”

Response. *Thank you!*

Action. *Fixed typo (which would have been on L385 of the revised m/s).*

Supplementary materials
S.1.4.3
In equation (S37) the first term on the right-hand side lacks a minus sign. The same holds for the following equalities, the first terms lack a minus sign.

Response. *Thank you!*

Action. *Fixed typo. in Eq. S37 and the equation directly below (two additional minus signs in the two equations on L265-268 of the Supplementary Information).*

S.1.4.4

It is not clear whether this paragraph concerns the case of negligible mortality, as μ does not appear in equation (S38). On the other hand, you refer later in line 241 to the specific case of μ=0.

Response. *No we were no longer considering the case of negligible mortality in this part of the Supplementary Information. However, we recognize this might have opaque since this section follows another in which there was no mortality. The confusion is not helped by the notation used for our scaling of the parameters, which incorporates the parameter* μ *into the scaled parameters* $\hat{\beta}$ *and* $\hat{\gamma}$*. We therefore have altered the relevant part of the Supplementary Information to remind the reader both that the context has changed and of this change in notation.*

Action. *Additional text (L271 and L274 of the Supplementary Information).*
